# scReQTL: an approach to correlate SNVs to gene expression from individual scRNA-seq datasets

**DOI:** 10.1186/s12864-020-07334-y

**Published:** 2021-01-08

**Authors:** Hongyu Liu, N. M. Prashant, Liam F. Spurr, Pavlos Bousounis, Nawaf Alomran, Helen Ibeawuchi, Justin Sein, Piotr Słowiński, Krasimira Tsaneva-Atanasova, Anelia Horvath

**Affiliations:** 1 McCormick Genomics and Proteomics Center, School of Medicine and Health Sciences, The George Washington University, Washington, DC 20037 USA; 2 Chinese Medicine Toxicological Laboratory, Institute of Traditional Chinese Medicine, Heilongjiang University of Chinese Medicine, Harbin, 150040 People’s Republic of China; 3 Department of Medical Oncology, Dana-Farber Cancer Institute, Boston, MA 02215 USA; 4 Cancer Program, The Broad Institute of MIT and Harvard, Cambridge, MA 02142 USA; 5 Pritzker School of Medicine, Biological Sciences Division, The University of Chicago, Chicago, IL 60637 USA; 6 Translational Research Exchange at Exeter, University of Exeter, Exeter, EX4 4QJ UK; 7 EPSRC Centre for Predictive Modelling in Healthcare, University of Exeter, Exeter, EX4 4QJ UK; 8 Department of Mathematics & Living Systems Institute, University of Exeter, Stocker Road, Exeter, EX4 4QD UK; 9 Dept. of Bioinformatics and Mathematical Modelling, Institute of Biophysics and Biomedical Engineering, Bulgarian Academy of Sciences, 105 Acad. G. Bonchev Str, 1113 Sofia, Bulgaria; 10 Department of Biochemistry and Molecular Medicine, Department of Biostatistics and Bioinformatics School of Medicine and Health Sciences, George Washington University, Washington, DC 20037 USA

**Keywords:** eQTL, ReQTL, scReQTL, single cell, VAF_RNA_, scVAF_RNA_, scRNA-seq, SNV, Genetic variation, RNA-seq, single cell RNA sequencing, single cell RNA-seq

## Abstract

**Background:**

Recently, pioneering expression quantitative trait loci (eQTL) studies on single cell RNA sequencing (scRNA-seq) data have revealed new and cell-specific regulatory single nucleotide variants (SNVs). Here, we present an alternative QTL-related approach applicable to transcribed SNV loci from scRNA-seq data: scReQTL. ScReQTL uses Variant Allele Fraction (VAF_RNA_) at expressed biallelic loci, and corelates it to gene expression from the corresponding cell.

**Results:**

Our approach employs the advantage that, when estimated from multiple cells, VAF_RNA_ can be used to assess effects of SNVs in a single sample or individual. In this setting scReQTL operates in the context of identical genotypes, where it is likely to capture RNA-mediated genetic interactions with cell-specific and transient effects. Applying scReQTL on scRNA-seq data generated on the 10 × Genomics Chromium platform using 26,640 mesenchymal cells derived from adipose tissue obtained from three healthy female donors, we identified 1272 unique scReQTLs. ScReQTLs common between individuals or cell types were consistent in terms of the directionality of the relationship and the effect size. Comparative assessment with eQTLs from bulk sequencing data showed that scReQTL analysis identifies a distinct set of SNV-gene correlations, that are substantially enriched in known gene-gene interactions and significant genome-wide association studies (GWAS) loci.

**Conclusion:**

ScReQTL is relevant to the rapidly growing source of scRNA-seq data and can be applied to outline SNVs potentially contributing to cell type-specific and/or dynamic genetic interactions from an individual scRNA-seq dataset.

**Availability:** https://github.com/HorvathLab/NGS/tree/master/scReQTL

**Supplementary Information:**

The online version contains supplementary material available at 10.1186/s12864-020-07334-y.

## Background

In recent years, single cell RNA-seq (scRNA-seq) has become an increasingly accessible platform for genomic studies [1]. By enabling cell-level analyses, scRNA-seq has major advantages for studying gene-regulatory relationships. Among others, the ability to distinguish cell populations and to assess cell-type specific transcriptome features, have shown great potential to identify new regulatory networks [2–4]. Furthermore, scRNA-seq enables the assessment of intracellular molecular relationships, which can reveal cell-specific gene-gene interactions and co-regulated genetic features [2, 5, 6]. These relationships can be reflected in mutually correlated molecular traits, including gene expression (GE) and expression of genetic variants, such as Single Nucleotide Variants (SNVs).

A popular method to study SNVs effects on GE is eQTL (Expressed Quantitative Trait Loci), which is based on testing for a correlation between the number of alleles bearing the variant nucleotide at the position of interest, and the level of local (cis) or distant (trans) GE [7]. eQTLs have been mapped by large-scale efforts such as Genotype-tissue Expression Consortium (GTEx), PsychENCODE, ImmVar BLUEPRINT, and CAGE [8–12].

Recently, pioneering eQTL studies on scRNA-seq data have emerged. By utilizing the advantages of the single cell resolution, these studies have revealed many new regulatory SNVs, including those with cell-specific or transient effects [2, 13–16]. To assess GE, these methods employ approaches specific to single cell transcriptomics, including accounting for drop-outs, classification of cells by type, and assessments of progressive cell stages [2–4, 13–16]. SNV information is traditionally obtained from the genotypes across multiple individuals and encoded as the number of alleles (0, 1 or 2) bearing the variant nucleotide. Accordingly, eQTL analyses are confined to SNVs present in a sufficient number of individuals in the studied group, and frequently exclude variants with low minor allele frequency in the population.

Here, we explore an alternative approach to assess the correlation between GE and expression of SNVs located within transcribed genes from scRNA-seq data. The expression of the SNVs is estimated as the proportion of variant-bearing RNA molecules (Variant Allele Fraction, VAF_RNA_) at biallelic SNV loci. To correlate VAF_RNA_ to GE from single cells, we estimate VAF_RNA_ in the individual cell alignments, and correlate VAF_RNA_ with GE from the individual cells using a linear regression model [17]. To develop the pipeline, we used recent methodologies for calling SNVs and VAF_RNA_ estimation from RNA-seq data [18–23], as well as scRNA-seq-specific methods to estimate GE [24]. We also adopted a strategy from a method recently developed in our lab to correlate VAF_RNA_ and GE from bulk RNA-sequencing data – ReQTL (RNA-eQTL) [25]. We term the application of this technique on single-cell RNA-sequencing data: scReQTL.

We applied scReQTL on publicly available scRNA-seq generated on the 10 × Genomics Chromium platform using 3′-based protocol on 26,640 human adipose-derived mesenchymal stem cells (ADSCs), obtained from three healthy donors. This scReQTL analysis includes approximately 4 billion scRNA-seq reads. ScReQTL analysis was performed after classification of the cells by cell type, and only SNVs covered by a minimum of 10 unique sequencing reads per cell were included in the analysis. Across the three samples, we identified 1272 unique scReQTLs. scReQTLs common between individuals or cell types were consistent in terms of the directionality of the relationship and the effect size. In addition, scReQTLs were substantially enriched in known gene-gene interactions and significant genome-wide association studies (GWAS) loci.

## Results

### Overview of scReQTL workflow

**Fig. 1:**
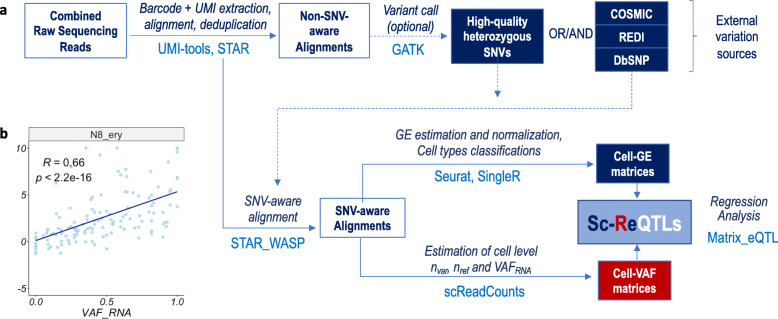
ScReQTL workflow (**a**), with an example of a significant scReQTL correlation between the SNV at 10:4977767_G > A and the gene *AKR1C1* **(b**)

An example of scReQTL workflow using publicly available tools is presented in Fig. 1 and outlined in detail in *Methods*. Below, we describe the workflow elements that we identified as specific and essential for the scReQTL analysis.

The scReQTL workflow includes three major components: scRNA-seq data processing, VAF_RNA_ assessment, and SNV-GE correlation by cell type.

*Processing* includes barcode and UMI modeling, alignment, GE estimation and cell type classification, and can employ a variety of publicly available tools. In the exemplified workflow, we process the barcodes using UMItools, and align using STAR the alignments are then deduplicated based in UMIs [26, 27]. Because VAF_RNA_ estimations can be sensitive to allele mapping bias, SNV-aware alignment recommended. We perform SNV-aware alignment for the list of positions of interest to be used as input for scReQTL analyses. Here, we perform SNV-aware alignment against the biallelic positions called by GATK in the corresponding pooled alignments applying a two-pass 2-pass STAR-WASP as previously described [18, 28, 29]. Alternatively, scReQTL can be applied on genomic positions of interest from external sources, for example sets of somatic mutations from the COSMIC database, or known RNA-edited loci from the REDI portal [30, 31]; in these cases, the selected sets of loci can be used as input for STAR-WASP alignment for.

**Fig. 2:**
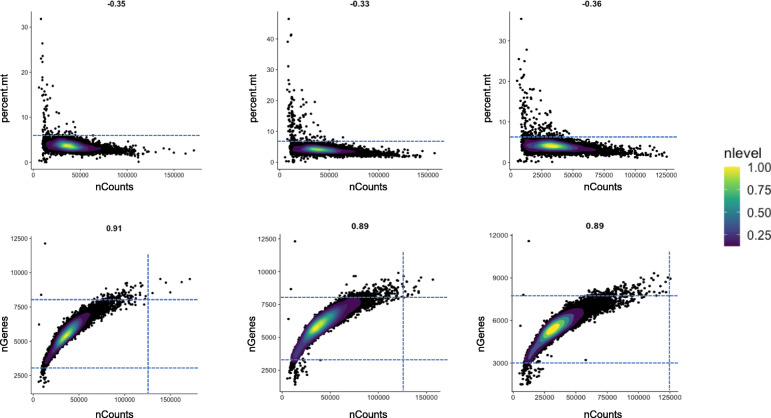
Density plots showing the distribution of cells based on proportion of transcripts of mitochondrial origin (top), and number of genes (bottom) plotted against the counts of sequencing reads in the three samples (from left to right: N8, N7, N5). The dotted line indicates the selected QC thresholds: mitochondrial gene expression above 6%, and number of genes below 3000. To remove potential doublets/multiples we also filtered out signals with more than 8000 genes and/or over 125,000 read counts

*GE estimation* is performed on the SNV-aware alignments, using FeatureCounts to assess the raw gene counts [32], followed by Seurat for normalization and GE variance stabilization [24, 33]. The generated GE expression values are then used to remove low quality data, batch effects and cell-cycle effects. The distributions of genes and RNA-seq reads, and the selected QC threshold are shown on Fig. 2. The effects of batch-correction and cell-cycle effects removal are shown on Supplementary Figure 1. On the high-quality cell-set we then apply Seurat [33], to normalize gene expression and to identify the most variable genes to be used in the scReQTL analyses (See Methods).

**Fig. 3:**
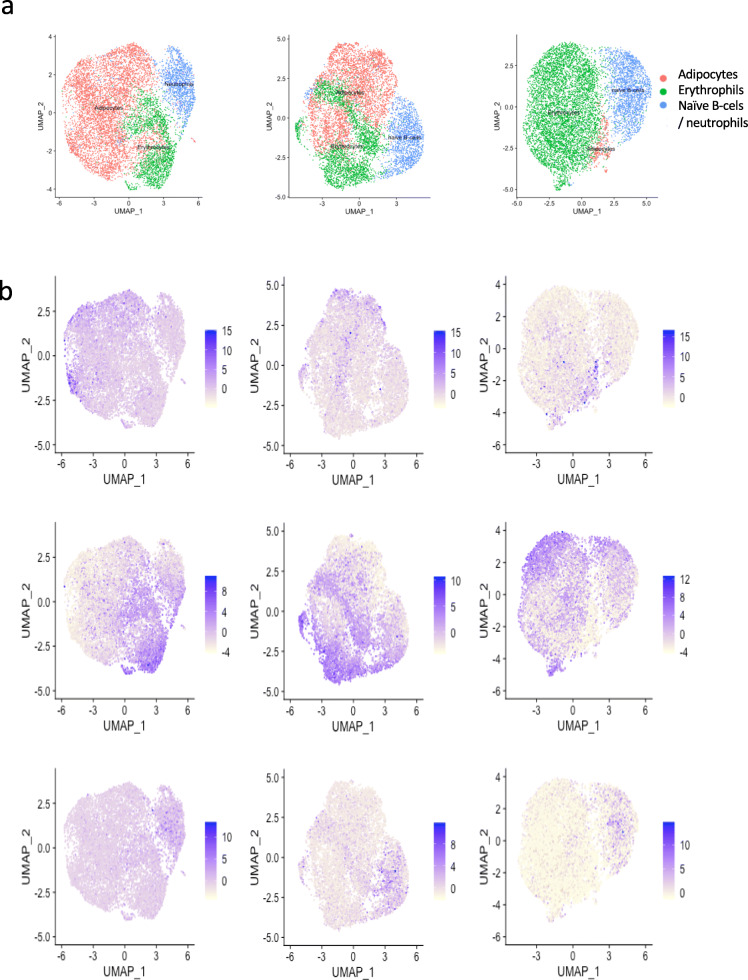
**a**) Cell types identified in each donor using SingleR. Adipose cells and erythrocytes were found in all three donors, whereas naïve-B-cells were seen in N5 and N7 and neutrophils only in N8. **b**) expression of genes associated with cell types: DCN (adipose cells, top), *H2AFZ* (erythrocytes, middle), and H1F0 (neutrophils and naïve B cells)

Cell type identification is performed using SingleR [34]. The expression profile of each single cell was correlated to expression data from the BluePrint + ENCODE dataset. Across the three study samples, four major cell types were identified: adipose cells, erythrocytes, neutrophils, and naïve-B cells. Adipose cells and erythrocytes were found in all three samples, whereas naïve-B cells were seen in N5 and N7 and neutrophils – in N8 (Fig. 3 and Supplementary Figure 2).

**Fig. 4:**
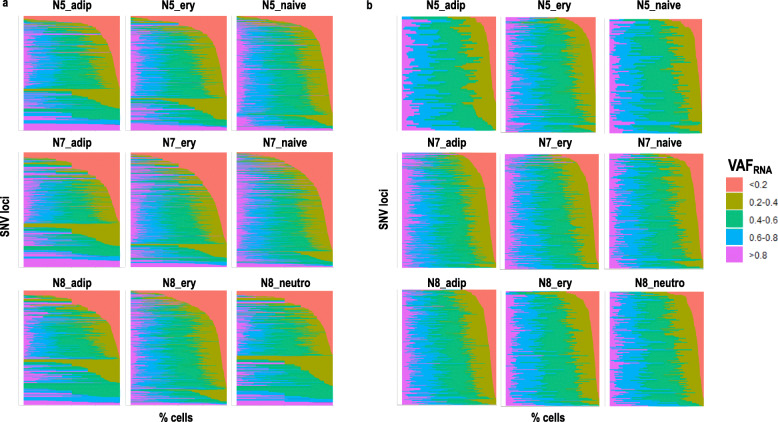
Distribution of scVAFRNA values estimated at SNV sites (displayed on the y-axis) with minR> 10 before (a) and after (b) filtering of non-variable SNV loci. The SNV sites are sorted by decreasing percentage of cells (x-axis) with scVAFRNA values < 0.2

*VAF*_*RNA*_ is assessed from the individual cell alignments at the positions of interest using SCReadCounts [35]. For each position, SCReadCounts estimates the number of sequencing reads bearing the variant and the reference nucleotide (n_var_ and n_ref_, respectively), calculates VAF_RNA_ (VAF_RNA_ = n_var_ / (n_var_ + n_ref_)) and outputs the values in an SNV-barcode matrix. The SNV-barcode matrices are in a format analogous to the GE-barcode matrices and can be directly used in the ReQTL analyses. To address stochasticity of sampling, estimations of VAF_RNA_ require a threshold of minimal number of unique sequencing reads (minR). Our previous research shows that current scRNA-seq datasets can contain hundreds of SNV sites covered by minimum of 10 sequencing reads (minR > 10) and thousands of SNV sites with minR > 5 [28]. In the herein presented analysis, we used VAF_RNA_ estimated at sites with minR > 10; from here on, we refer to these loci as informative. We note that for minR we are referring to sequencing reads with unique UMIs which are derived from unique mRNA molecules. The VAF_RNA_ distribution of the qualifying SNVs is then examined to identify the most variable VAF_RNA_ loci (see Methods). VAF_RNA_ distributions before and after filtering of uninformative (minR< 10) and non-variable VAF_RNA_ are shown on Fig. 4a and b, respectively.

*SNV-GE correlations* (scReQTLs) are then computed for each donor, stratified by cell type (see Methods). To qualify for scReQTLs analysis an SNV locus is required to have informative and variable VAF_RNA_ estimations from at least 20 cells per analysis. The variable VAF_RNA_ were correlated to the normalized GE values of the variable genes using linear regression model as implemented in Matrix eQTL [17]; quantile-quantile plots (QQ-plots) are presented on Supplementary Figure 3. Cis- and trans-correlations were annotated as we have previously described for the bulk ReQTLs [25]. Briefly, because scReQTLs are assessed from transcripts, we assign cis-correlation based on the co-location of the SNV locus within the transcribed gene; all the remaining correlations are annotated as trans-scReQTLs).

### Overall scReQTL findings

**Fig. 5:**
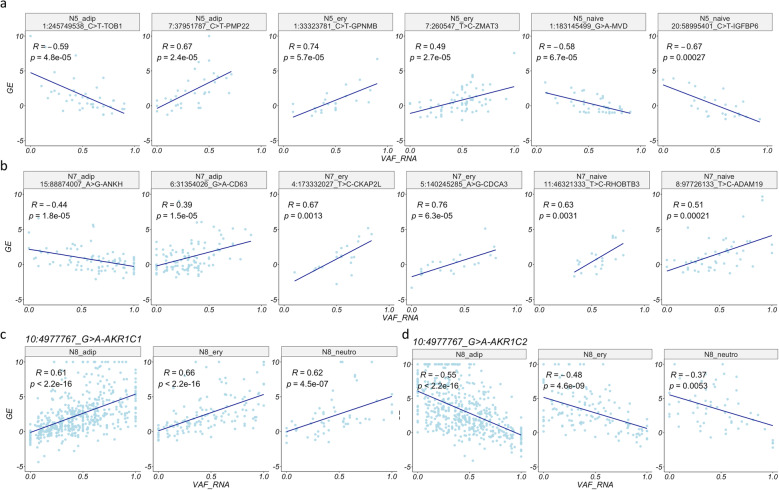
Examples of significant (FDR = 0.05) scReQTL correlations in donor N5 (a), N7 (b) and N8 (c and d). In N8, consistent across the three cell types cis-scReQTL is shown between the SNV at 10:4977767_G > A and its harboring gene *AKR1C1* (c), and between the same SNV and the nearby positioned gene *AKR1C2* (trans-scReQTL, d). Note that the displayed *P*-values are calculated based on the input for the plots generated using the R-package ggplot2 and do not represent the FDR—corrected values from the scReQTL analysis performed with Matrix eQTL

The number of variable genes and VAF_RNA_ loci retained for scReQTL analysis in the three donors (by cell type) is shown in Table 1. We performed scReQTL analysis separately for each individual and cell type; accordingly, 9 scReQTL analyses were run. Among the samples and cell types, between 79 and 316 SNV loci, and between 2114 and 2442 genes were used as input for scReQTL analysis. Across the 9 groups, a total of 644 distinct SNVs and 2571 distinct genes were tested. This analysis identified 1272 unique scReQTLs at false discovery rate (FDR) of 0.05. All significant scReQTLs are listed in Supplementary Table 1; examples are shown on Fig. 5.

**Table 1:**
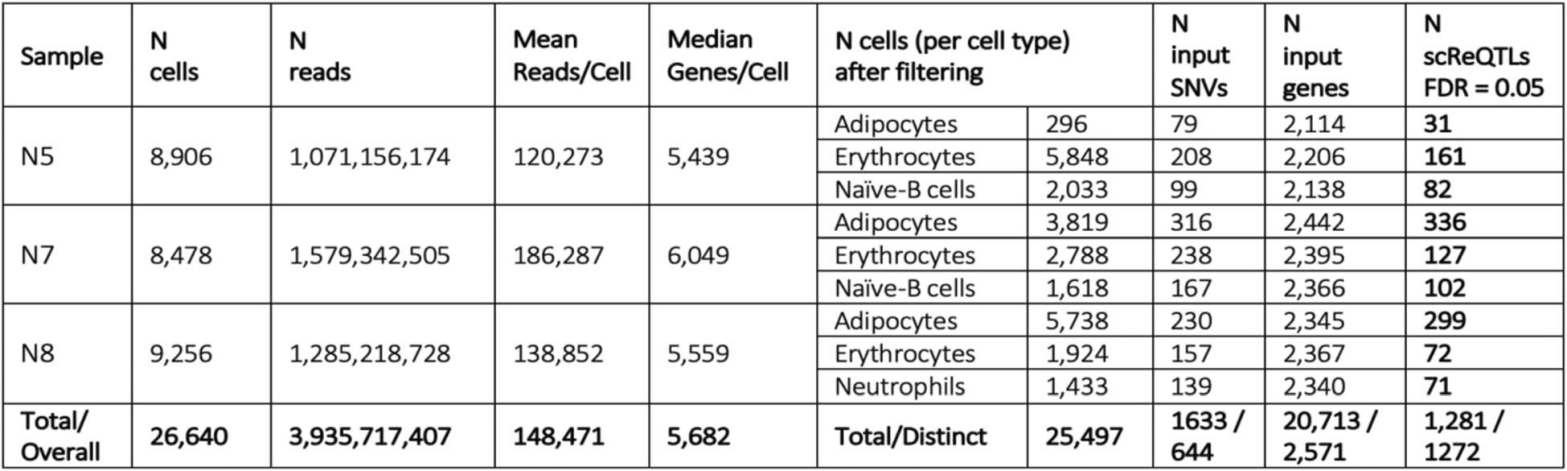
Input parameters for scReQTL analysis, and number of identified scReQTLs per cell type

Among the unique scReQTLs, 7 were identified in more than one cell type or sample (Supplementary Table 2). In all these cases, the correlations were in the same direction, and the effect sizes were similar (See Fig. 5c and d). We note that the number of common input-SNVs across the 3 samples was as low as 20 (numbers of common input SNVs and genes, as well as the common scReQTLs SNVs and genes are shown in Supplementary Figure 4).

**Fig. 6:**
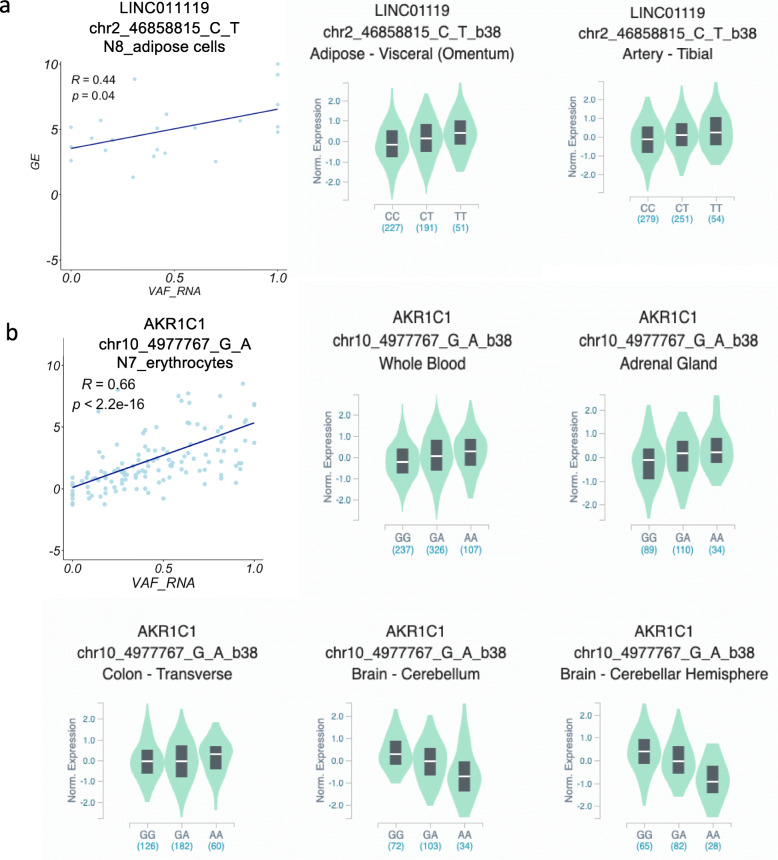
**a** Left: scReQTL between the SNV at 2:46858815_C > T and its harboring gene *LINC01119* (cis-scReQTL) identified in adipose cells from ADSC and compared to eQTLs from bulk adipose (middle) and bulk artery tibia (right) datasets; the bulk eQTLs are obtained from the GTEX database. The graphs are generated at the GTEx portal (https://www.gtexportal.org/). The eQTLs and scReQTL agreed in terms of directionality and effect sizes. **b** scReQTL between the SNV at position 10:4977776 and its harboring gene *AKRIC1* in ADSC-derived erythrocytes from ADSC (left-top) in a comparison with eQTLs from bulk RNA-seq data obtained from a panel of tissues (GTEX). The scReQTLs from the erythrocytes agreed in terms of directionality and effect sizes with eQTLs from whole blood, adrenal gland, and colon

Next, we investigated the relationship between cis- and trans-scReQTLs. Of the significant scReQTLs, only 6 represented cis-correlations, representing 4 distinct SNV-gene pairs, (examples shown in Fig. 5c and Fig. 6). This low proportion of cis-scReQTL correlations differs from eQTL analyses, which typically identify a high number of significant cis-correlations and is attributed to several factors. First, in contrast to the eQTL distance-based cis/trans annotation, scReQTL employs gene-based annotation, which results into a cis-to-trans shift for SNVs in nearby genes. Second, cis-scReQTL estimations require a certain level of expression of the SNV-harboring genes (as defined by minR) thereby confining cis-scReQTL analyses to moderately-to-highly expressed genes in the system. Third, the scReQTL input SNVs are confined to expressed regions, and in the herein employed system (10xGenomics Chromium 3′-protocol) most of them are 3′-UTR-located. Indeed, two of the cis-scReQTL SNVs were located in the 3’UTR of their harboring genes, one was a synonymous substitution (G246G in *TNNT3*) and one was located in an exon of the non-coding RNA *LINC01119*. In contrast, cis-eQTLs are frequently located in the promoter and other regulatory sequences, often transcriptionally silent and therefore not detected by the ReQTL/scReQTL approach.

To further investigate the connection between cis- and trans-scReQTLs, we assessed if some scReQTLs are mediated by cis-effects that do not reach significance at an FDR of 0.05. To do this, we computed the correlation of all SNVs represented in significant trans scReQTLs with their harboring gene. For 26% of the scReQTL SNVs, we detected correlations with their harboring genes with 0.05 < FDR < 0.1 (Supplementary Figure 5). This analysis suggests that a proportion of the SNVs may at least partially exert their trans-effects via weak to moderate regulation of the expression of their harboring gene.

### scReQTL in known genetic networks

To assess to what extend scReQTL findings agree with known SNV-gene, and gene-gene interactions, we intersected the significant scReQTLs with: (a) eQTLs reported in the GTEx database [8], (b) ReQTLs as estimated from bulk adipose sequencing data [25], (c) known gene-gene interaction from the STRING database [36], and (d) significant GWAS loci [37].

#### scReQTLs and eQTLs from GTEx

To estimate the overlap between scReQTL and known eQTLs, we used the data from 53 different tissues and cell types from the GTEx database (https://www.gtexportal.org). First, we identified the SNVs and genes used as an input for scReQTLs, and participating in known eQTLs: a total of 111 input SNVs and 2024 input genes participated in at least one eQTL reported in GTEx. Across the 49 tissues, scReQTL identified 32 correlations (Supplementary Table 3), comprised of 6 unique SNV-gene pairs (5 SNVs and 6 genes). These pairs included all 4 significant cis-scReQTLs, and two trans-scReQTLs: chr10_4,977,767_G > A and *AKR1C2* (see Fig. 5d), and chr1:115337511_G_A and *NGF*. For each of the 6 SNV-gene pairs, we compared the scReQTLs and the eQTLs in the different GTEx tissue types. For 3 of the 6 scReQTLs, the corresponding GTEx eQTLs were consistent in terms of directionality and effect size (Fig. 6 and Supplementary Figures 6 and 7).

The other 3 scReQTL were found as both positive and negative eQTLs depending on the tissue type in GTEx. The positive cis-scReQTL, chr6:31354105_G > A_HLA-B, was a significant cis-eQTL in 4 GTEx tissues: positive in three, but negative in the testis (Supplementary Figure 8). The last 2 scReQTLs comprised correlations of the SNV at chr10:4977767_G > A with *AKR1C1* (positive) and *AKR1C2* (negative); these scReQTLs were consistent across cell types (see Fig. 5c and d). In GTEx, the corresponding eQTLs were found in multiple tissues, and in both positive and negative correlations, highlighting tissue-specific effects (Supplementary Figures 9 and 10).

Overall, our analysis on the agreement between significant scReQTLs and eQTLs identified a narrow overlap, within which most observations were consistent, and the remaining were not contradictory. We note that this analysis was limited by the relatively small number of input scReQTL SNVs present in GTEx. Furthermore, the majority of the significant scReQTLs were in trans, which are known to be highly tissue-specific [8]. None of the 4 cell types assessed in our study - adipose cells, erythrocytes, neutrophils, and naïve-B cells obtained from ADSCs - were a direct match to any of the 49 tissues and cell types for which eQTLs were available from the GTEx database. Finally, we expect that the strongest contributor to the low overlap between scReQTL and eQTLs is the detection power of scReQTL. Specifically, depending on the sequencing depth per cell, many cells do not pass the minR requirement for a given SNV (especially at minR> 10, which is the cutoff used here), and are therefore excluded from the analysis. Indeed, while the initial cell counts per scReQTL analysis (except for N5 adipose cells) were over 1000, the majority of the SNV loci were expressed in between 20 (the required minimum) and 100 cells with minR> 10 per cell type (Supplementary Figure 11a). In comparison, the GTEx eQTLs are computed from a minimum of 100, and in most of the tissues, from over 250 individuals (Supplementary Figure 11b).

#### scReQTLs and ReQTLs from bulk adipose tissue

**Fig. 7:**
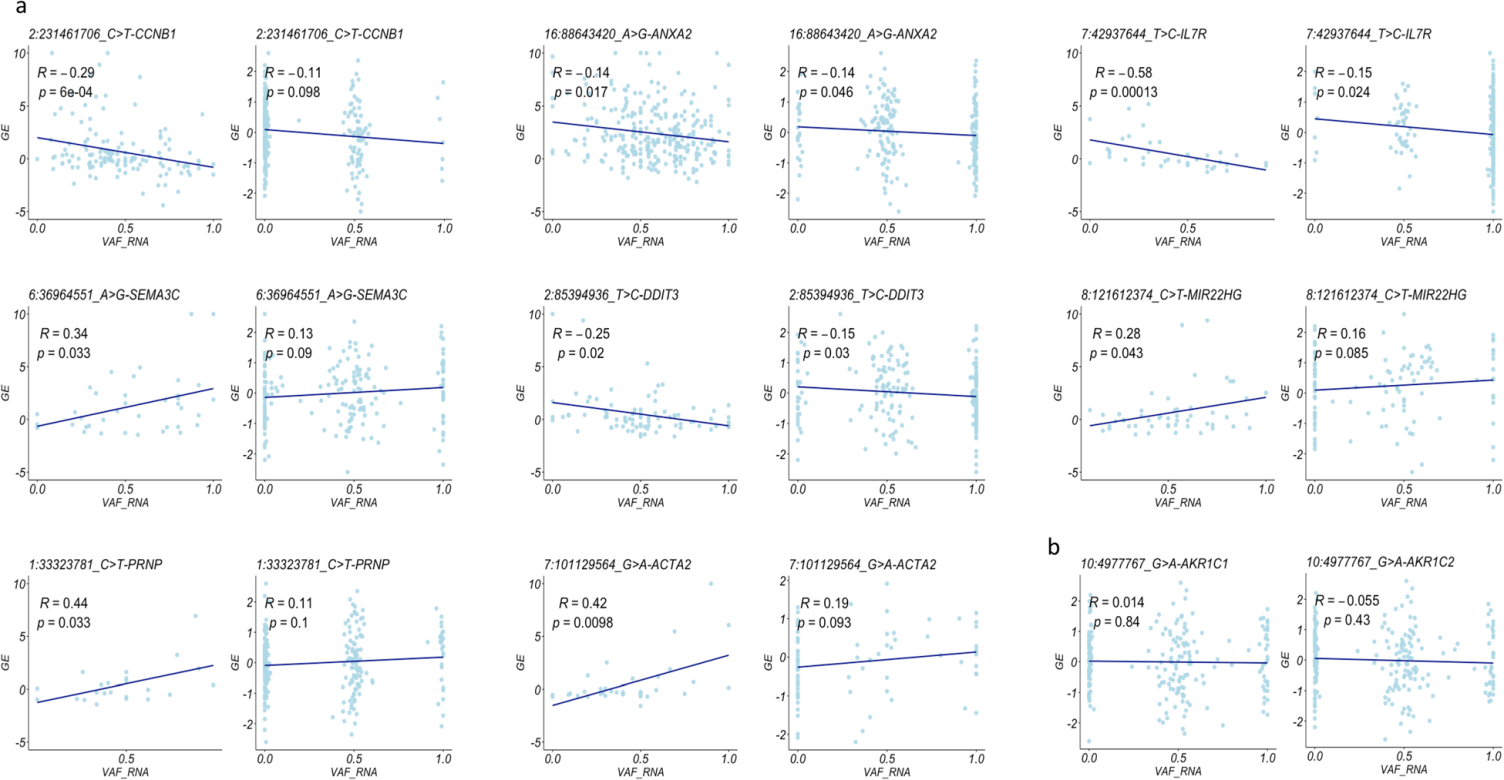
scReQTLs and ReQTLs from bulk adipose tissue. **a** Examples of comparisons of scReQTLs (left) and ReQTLs from bulk adipose tissue (right) at FDR = 0.1. The ReQTLs had generally weaker size effects and agreed in directionality in 71% of the correlations. Note that the displayed P-values are calculated based on the input for the plots generated using the R-package ggplot2 and do not represent the FDR—corrected values from the scReQTL analysis performed with Matrix eQTL. **b** ReQTL analysis between the SNV at 10:4977767 and *AKR1C1* (left), and *AKR1C2* (right), which were found as significant scReQTLs, did not show significant correlation in bulk RNA-seq data

Next, we intersected the scReQTL findings with the ReQTLs from bulk RNA-sequencing data. To do this, we performed ReQTL on RNA-seq data from two adipose tissues downloaded from GTEx – adipose subcutaneous (275 samples) and adipose visceral (215 samples) - following the published protocol [25]. Using the same SNVs and genes used as input for the scReQTL, with an FDR = 0.05, ReQTL did not identify significant correlations, whereas with an FDR = 0.1, ReQTL identified 84 (6.6%) and 48 (3.8%) of the significant scReQTLs, in adipose subcutaneous and visceral tissue, respectively. The majority of these ReQTLs had small effect sizes and agreed in the direction with the corresponding scReQTL in 71% of the cases (Examples shown on Fig. 7a). Of note, the above discussed chr10:4977767_G > A and *AKR1C1*/*AKR1C2* did not show any correlation when examined from bulk RNA-seq data (Fig. 7b).

The different sets of SNV-gene pairs identified by scReQTL and ReQTL (as well as eQTL) suggests different regulatory relationships captured by scReQTLs. Bulk ReQTLs and eQTLs show a high overlap between each other and are both based on abundance of variant alleles across multiple individuals with different genotypes. In contrast, scReQTL operates in a setting of identical genotypes, where it is likely to capture RNA-based genetic interactions, possibly with transient and/or cell-specific effects.

#### scReQTLs and known gene-gene interactions

**Fig. 8:**
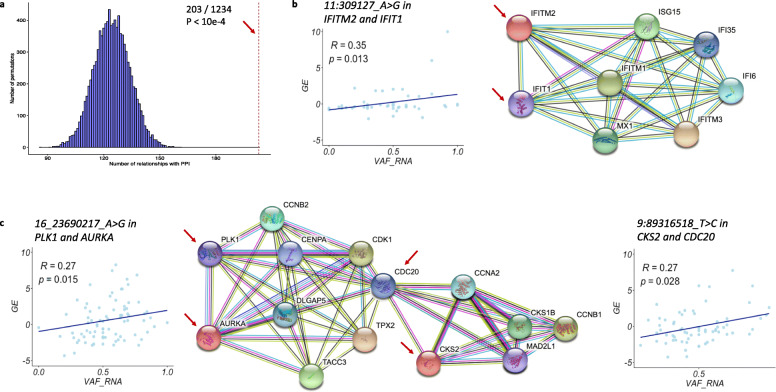
**a** Permutation test for assessment of enrichment of trans scReQTLs in known gene-gene interactions obtained from the STRING database; 10,000 permutations were used. The *p*-value (*p* < 10e-4) was defined as the fraction of permutations in which the number of gene-gene pairs found in the known interaction database was at least as great as the number found in the observed data. This analysis showed significant enrichment of trans-scReQTLs with known gene-gene interactions. b and c) Examples of trans-scReQTLs and known gene-gene interactions: *IFITM2* (11:309127_A > G) in and *IFIT1* (**b**) and *PLK1* (16_23690217_A > G) and *AURKA*, and *CKS2* (9:89316518_T > C) and *CDC20* (**c**). The interaction graphs are generated using the STRING database visualization tools. Note that all the scReQTL highlighted gene-gene interactions are supported by a minimum of three lines of evidence that include either experimental validation (purple line) or curated databases (light-blue line), or both

Because the vast majority of the significant scReQTLs were in trans (i.e. representing correlations between two different genes, VAF_RNA_ of an SNV located in one of the genes and expression level of the other), we assessed if these gene pairs were enriched in known gene-gene interactions. We downloaded the known gene-gene (human) interactions from the STRING database [36] and intersected these with the scReQTLs. From the 1234 unique gene-gene scReQTLs pairs, 203 (16.4%) were previously annotated in STRING (Supplementary Table 4, *p* < 10e-4, permutation test using 10,000 permutations, Fig. 8a). Examples include *IFIT1* and *IFITM2*, *AURKA* and *PLK, and CKS2* and *CDC20* (Fig. 8b-c). The strong enrichment of scReQTLs with known genetic networks suggests that scReQTLs may be used to identify allele contributions to gene-gene interactions.

#### scReQTLs and GWAS

Furthermore, we intersected the SNVs participating in scReQTLs with SNVs significantly associated with phenotypes by GWAS [38]. This analysis showed that 18 (out of the 408 unique scReQTL SNVs, 4.4%) were present in GWAS; these 18 SNVs participated in 84 scReQTL correlations (Supplementary Table 5). This percentage is slightly higher than the overlap between GWAS and GTEx eQTLs (3.7 and 3.6% in adipose visceral and adipose subcutaneous tissue respectively), and significantly higher than the overlap with common SNVs from DbSNPv.154, (0.34%, p < 10e-6). This analysis shows that scReQTL SNVs are enriched in genetic variants associated with phenotype via large population-based and case-control studies.

### Functional scReQTLs SNVs annotations

We assessed the SNVs participating in scReQTL in regard to position in the harboring gene and the predicted functional effects. As expected from scRNA-seq data generated using a 3′-based protocol, the majority of the SNVs resided in the 3’UTR of their harboring gene (70.2%, Supplementary Figure 12); the 3′-UTR SNVs participated in 69.6% of the scReQTLs. 3′-UTR variants are known to strongly affect both GE levels and splicing [39–42]; hence, scReQTLs can be applied to study these aspects of genetic regulation. The second category was exonic SNVs, comprising 16.2% of the unique SNVs and participating in 14.9% of the scReQTLs. Exonic SNVs included missense, nonsense, and near-splice variants, many of which can potentially affect the protein structure and function. Of note, scReQTL captured a substantial number of intronic SNVs – 13%, participating in 11.2% of the scReQTLs. Intronic sequences are reported in 15–25% of the RNA-sequencing reads from both bulk and single-cell RNA-seq [4, 41, 42]. Intron quantitation can be used to estimate the relative abundance of the precursor and mature mRNA, thereby assessing the RNA velocity and dynamic cellular processes [4]. In the allele-specific setting provided by the scReQTLs, the correlations of intronic SNVs with GE can identify SNVs regulating the RNA splicing.

Next, we assessed if the scReQTLs SNVs are enriched in specific clinical phenotypes obtained from the ClinVar database [43]. Fifteen SNVs (3.7% of the total 408 distinct scReQTL SNVs) were associated with known clinical phenotypes, including circulating phospholipid trans fatty acids, cortisol levels, circadian rhythm, risk for cardiovascular disease, blood pressure, schizophrenia, neuroticism, osteoporosis, anthropometric traits, and asthma (See Supplementary Table 1). This percentage is similar to the overlap between ClinVar and GTEx eQTLs (3.3 and 3.1% of the eQTLs in adipose visceral and adipose subcutaneous tissue respectively), and significantly higher than the overlap with common SNVs from DbSNPv.154, (0.61%, *p* < 10e-6). Finally, we assessed the predicted functional and/or pathogenic scores of the scReQTL SNVs using 17 models including SIFT, Polyphen2, LRT, MutationTaster, MutationAssessor, FATHMM, PROVEAN, VEST3, CADD, DANN, fathmm-MKL, MetaSVM, MetaLR, integratedFit, GERP++, phyloP, and phastCons, as implemented in ANNOVAR [44]; this data is summarized in Supplementary Table 6).

### scReQTL application

Application of scReQTLs requires consideration of several factors. First, because scReQTLs assess expressed SNV loci, they cannot capture variants in transcriptionally silent genomic regions. In addition, SNV loci with expression levels below the required minimum number of RNA-seq reads (minR) are not included in the scReQTL analyses. Furthermore, when assessed with the platform used in this study - 10x Genomics Chromium v3 chemistry – the analyzed SNVs are confined to those located within the length of the sequencing read (here, 150 nt) from the 3′ end of the transcript. For the above reasons, scReQTLs accessible SNVs represent a subset of the expressed SNVs and are not designed to cover the full set of SNVs in the genome/transcriptome.

Second, when a genetically regulated gene is captured by scReQTL analysis, the scReQTLs may highlight SNVs that are co-allelic to the actual causative SNV(s). This is the case for SNVs positioned outside the transcribed regions or outside the coverage of the sequencing library.

Third, scReQTLs are based on VAF_RNA_ estimation, which can be affected by technical parameters, including allele mapping bias [45, 46]. Therefore, we perform the scReQTL using SNV-aware alignments. Specifically, we apply STAR-alignment with WASP, which removes ambiguously mapped reads after checking for consistency with the reads containing the alternative nucleotide [27, 29].

Another important parameter for VAF_RNA_ estimation is the selection of cutoff for minimal number of reads, minR. When selecting minR for an analysis, a major factor is the balance between the confidence of VAF_RNA_ estimation (high minR) and the inclusivity of SNVs (lower minR values include more loci for scReQTL). Our previous research shows that for current 10× Genomics scRNA-seq datasets, minR > 5 provides a reasonable balance between VAF_RNA_ confidence and SNV inclusivity, while at lower cutoffs (i.e. minR = 3) stochasticity of sampling can affect the VAF_RNA_ estimation [28]. In the present study, we have included SNV loci with minR > 10. In addition, to assess the scReQTL at lower minR cut-offs, for a subset of the dataset (N7) we performed repeated analyses varying the value of minR in one-step increments between 5 and 9 and analyzed the outputs. The first observation is that, as expected, minR inversely correlates with the number of input SNV loci and the number of significant scReQTLs (Supplementary Figure 13a). Second, the outputs of the scReQTLs show only partial overlap across different minR cut-offs, which is due to the partial overlap between the input SNVs (Supplementary Figure 13b-d). We reason that the partial overlap in the input SNVs loci is largely due to the very stringent filtering criteria applied to retain the loci with the most variable VAF_RNA_. Specifically, we filter out loci for which over 75% of the VAF_RNA_ values are in the range of 0.5 ± 0.1 (corresponding to stable biallelic expression; this removed more than 50% of all loci), as well as loci with over 75% of the VAF_RNA_ values in the ranges 0–0.25 or 0.75–1 (corresponding to predominantly monoallelic or skewed allelic expression). As we show in our previous research [28], the VAF_RNA_ distribution changes substantially with different minR, which affects the subset of variants retained after filtering. Importantly, each of the distinct sets of significant SNV-gene pairs obtained at different minR showed very strong enrichment in known gene-gene interactions, which supports confident scReQTL observations with VAF_RNA_ cutoffs of 5 and above (Supplementary Figure 14).

Another threshold to consider is the minimal number of cells to be used for scReQTL, in this study set to 20. Using a minimum of 20 cells for assessing SNV-GE correlations is a result of setting a threshold for filtering loci with non-variable VAF_RNA_. Specifically, the locus is considered to be variable if in at least 15 (75%) out of the 20 cells VAF_RNA_ is in the range 0.25–0.4 or 0.6–0.75 (i.e we exclude stable biallelic, monoallelic, and skewed allelic expression). From the binomial distribution we compute that this gives 0.0206 probability of observing variable loci by chance assuming a 50% probability of success on each of at least 15 out of 20 trials. This probability decreases with increasing number of cells. We consider the maximal 0.02 chance threshold for wrongly assigning variability of the loci to be reasonably conservative and acceptable. In addition, we showed in our recent study [23] that 20 VAF values are sufficient to model the characteristics of VAF distributions and that higher numbers will improve the estimates (see Fig. 4 in [23]). For 10 cells, the probability of observing a single variable locus by chance would increase to 0.054 (scReQTLs from 10 to 19 cells are shown on Supplementary Figure 15).

Furthermore, scReQTLs can be affected by the accuracy of the variant call, including assessment of presence or absence of an SNV, and assignment of a biallelic state. The presented pipeline uses scRNA-seq data only, where we call SNVs from pooled scRNA-seq data and select for scReQTL analysis highly confident heterozygous sites based on mapping and Phred quality, genomic position (genic, non-repetitive regions), and previously validated rsIDs. To confidently assign biallelic state, we select SNVs with a minimum of 50 unique reads supporting each allele from the pooled scRNA-seq. By default, this selection excludes heterozygous SNVs with strong non-random monoallelic expression. Therefore, while the above approach is suitable for datasets with no matched DNA, when available, DNA-estimated genotypes can be helpful to interpret the context of the scReQTL findings.

Importantly, scReQTLs do not necessarily require variant calls and can be run on custom pre-defined lists of genomic positions such as a database of somatic mutations or RNA-editing sites. In this case the VAF_RNA_ is estimated for all the input sites, and SNVs not present in the assessed sample (i.e. SNVs with VAF_RNA_ = 0 across all cells) are removed during the VAF_RNA_ filtering step (see Fig. 4), while the remaining VAF_RNA_ estimations are used in the scReQTL analysis.

Finally, VAF_RNA_ varies between different cell types, often due to cell-specific regulatory mechanisms [47]. Due to the dynamic nature of RNA transcription, it is expected that VAF_RNA_ (similarly to GE) will vary depending on conditions, disease states and stochastic factors. Therefore, scReQTLs are expected to be transient and their interpretation requires consideration of the dynamics of the variables underlying the correlation.

## Discussion

Single-cell RNA-seq eQTL analyses define an emerging research niche that brings major benefits for the understanding of functional genetic variation including the identification of cell-type and condition-specific correlations [2, 13–16, 48]. In this paper, we present a new eQTL-based analysis in a scRNA-seq setting - scReQTL – which uses the VAF_RNA_ at expressed heterozygous SNVs in place of the genotypes, to correlate allele prevalence to gene-expression levels. By using VAF_RNA_ across multiple cells of the same sample, scReQTLs introduce several new analytical aspects.

First, and perhaps most importantly, as scReQTL is implemented on multiple single cells from the same sample, it can be applied to assess the effects of SNVs in a single sample or individual. This is particularly applicable for rare SNVs which are challenging to study via population-based approaches. We envision that this scReQTL feature can benefit studies on functionality of infrequent and de novo mutations causing rare phenotypes, as well as somatic mutations in cancer. Second, scReQTLs increase the dynamicity of the SNV-gene correlations, as VAF_RNA_, similarly to GE, is both dynamic and cell-type-specific [47]. In particular, in each cell type, scReQTL correlates the most variable VAF_RNA_ to the most variable genes. Third, as compared to the discrete genotype values (0,1,2), VAF_RNA_ can obtain continuous values spread along the entire VAF_RNA_ range ([0,1]), allowing for more precise computation of the proportion of each allele represented in the RNA in a given cell. Fourth, scReQTL operates in the context of (largely) identical genotypes, which narrows the observed effects to RNA-mediated interactions. Finally, scReQTL does not necessarily require matched DNA (although we recommend it for genotyping of heterozygous SNVs, if available), and therefore can be applied on scRNA-seq data alone. Related to that, scReQTL analyses can be performed using pre-defined SNVs of interest, such as RNA-editing sites and sets of dbSNP.

At the same time, compared to single cell and bulk eQTLs, scReQTL analyses have certain limitations. First, the scReQTL accessible SNVs are restricted by depth of coverage per cell (minR) and, in the case of 3′-based scRNA-seq protocols, by the length of the sequencing read. Therefore, scReQTLs can analyze only a proportion of the transcribed SNVs. This limitation is expected to be gradually reduced with the progress of the sequencing technologies. Additional attenuation of this constraint is possible through reducing the value of minR used in the analysis. Indeed, while in this study we apply minR > 10, which retained between 308 and 721 input SNVs per sample, in our prior research we show that at minR > 5 the number of SNVs is higher by an order of magnitude [28]. Second, scReQTL appears to have relatively low power to detect cis-acting (on the same gene) SNVs (See Supplementary Figure 3). Specifically, the vast majority of the correlations identified in this study are trans-scReQTLs. Several factors may account for this observation. As mentioned earlier, the definition of “cis”-scReQTLs is based on residing of the SNV within the same gene; hence SNVs that would be classified as “cis” using the eQTL distance-based definition are “trans” for the scReQTLs, increasing the proportion of trans-correlations in the same SNV-gene dataset. Additional possible explanation is that in the explored setting of minR> 10, cis-acting SNVs are located in genes with high expression, which likely contain a high proportion of stably expressed genes, including with house-keeping functions. Notably, the identified trans-scReQTLs are significantly enriched in known gene-gene correlations (See Fig. 7), therefore we interpret them as indictive of an allelic contribution to these gene-gene interactions. The above factors at least partially account for the narrow overlap between scReQTLs and eQTLs/ReQTLs. At the same time, scReQTLs are able to capture correlations that are masked in the bulk eQTL and ReQTL analyses (See Fig. 8).

Finally, at present, a direct comparison between scReQTLs and single cell eQTLs is limited, to a large extend due to a narrow overlap between the sets of SNVs matching the requirements for scReQTL inputs; these SNVs are located mostly in the expressed 3′-end of the gene, as compared to the genome-wide DNA-genotyped loci used in the sc-eQTLs. Additional contributing factors are the different cell sources as well as the different capturing protocols (i.e. SmartSeq vs 10xGenomics). With the advances of the scRNA-seq technologies, and the extension of single-cell QTL-based approaches to more tissues and cell types, comparisons between sc-eQTLs and scReQTLs are expected to provide meaningful information on underlying mechanisms in a cell-type specififc context.

Our scReQTL analysis includes approximately 4 billion RNA-seq reads from 26,640 human ADSCs, obtained from three healthy donors. We chose the 10xGenomics platform due to its growing popularity, high throughput, and the support for unique molecular identifiers (UMI) for the removal of PCR-related sequencing bias. Using stringent cutoff for SNV coverage (minR> 10) we identified 1272 distinct scReQTLs. These scReQTLs include a considerable number of correlations which involve SNVs previously highlighted by GWAS and are significantly enriched in known gene-gene interactions. These results demonstrate that scReQTLs can be used to identify novel genetic interactions, including those which are specific to a given cell-type.

## Conclusion

We present a new approach – scReQTL – that correlates SNVs to gene expression from scRNA-seq data. ScReQTL is relevant to the rapidly growing source of scRNA-seq data and can be applied to outline SNVs potentially contributing to cell type-specific and/or dynamic genetic interactions from an individual scRNA-seq dataset.

## Methods

### Data

We used publicly available scRNA-seq data [49] from 26,640 human cells from three healthy donors: N5, N7 and N8. The scRNA-seq data was generated on 10x Genomics Chromium v2 platform; the library preparation and sequencing are described in detail elsewhere [49]. Briefly, cells were partitioned using 10x Genomics Single Cell 3′ Chips, and barcodes to index cells (16 bp) and transcripts (10 bp UMI) were incorporated. The constructed libraries were sequenced on an Illumina NovaSeq 6000 System in 2 × 150 bp paired-end mode.

### SNV-aware alignment

The cell barcodes and UMIs were extracted using UMI-tools from the pooled (per donor) raw sequencing reads [26]. The pooled sequencing reads were aligned to the latest version of the human genome reference (GRCh38, Dec 2013) using STAR v.2.7.3.c in 2-pass mode with transcript annotations from the assembly GRCh38.79 [27]. The alignments were deduplicated retaining the reads with the highest alignment scores [26]. SNVs were called in the pooled deduplicated alignments using GATK v.4.1.4.1 [18]. To identify heterozygous SNV positions qualified for VAF_RNA_ analysis, we applied a series of filtering steps. Specifically, heterozygous SNVs were selected based on the presence of minimum of 50 high-quality reads supporting both (reference and alternative) nucleotides in the pooled alignments. SNV loci were annotated using SeattleSeq v.13.00 (dbSNP build 153), and loci positioned in repetitive or intergenic regions were removed. The SNV lists were further filtered based on the following requirements: QUAL (Phred-scaled probability) > 100, MQ (mapping quality) > 60, QD (quality by depth) > 2, and FS (Fisher’s exact test estimated strand bias) = 0.000. The filtered SNV lists (per donor) were then used as an input for a second, SNV-aware alignment using STAR-WASP [29].

### Gene expression estimation

To estimate gene expression, we first apply FeatureCount on the individual alignments to assess the row gene counts per cell [32]. We then normalize and scale the expression data using the *sctransform* function as implemented in Seurat v.3.0 [24, 33], which stabilizes the GE variance using regularized negative binomial regression, and outlines the most variable genes. The *sctransform* function integrates the previous Seurat functions NormalizeData, ScaleData, and FindVariableFeatures. The cell-feature distributions were than plotted to identify and filter out outliers and low-quality cells, which we defined as [1] cells with mitochondrial gene expression over 6%, cells with less than 3000 genes, and 3) cells with more than 8000 detected genes or > 12,500 UMI counts, (to remove potential doublets), as well as cells with mitochondrial genes’ expression higher than 6% of the total gene expression, and to correct for batch- and cell-cycle effects (See Fig. 2). We then integrate the datasets and use the function FindIntegrationAnchors to identify ‘anchors’ between pairs of datasets. This analysis resulted in 4099 common genes across the three samples, which we used to correct for batch effects. Next, we split the individual matrices by cell type, and for each cell type, genes which expression in 80% or more of the cells was within 20% or less from the top or bottom of the GE range, were filtered out. This retained between 2114 and 2442 per sample for scReQTL analyses. In addition, after examining the GE distribution across the cells (per cell type), the retained most variable genes were then used for scReQTL analyses (See Table 1).

### Cell type identification

To define individual cell types from the ADSCs, we used SingleR version 1.0.5 [34]. SingleR assigns cellular identity by comparison to reference whole transcriptome expression data sets of pure cell types. SingleR correlates the expression profile of each single cell to whole-transcriptome expression data from established cell types (BluePrint + ENCODE datasets). To select the expression profile most similar to the tested cells, the analysis is rerun iteratively, using only the top cell types from the previous step until only one cell type is retained. Comparing our datasets against 259 bulk RNAseq profiles representing 24 main cell types and 43 subtypes, SingleR identified four major cell types: adipose cells and erythrocytes were found in all three samples, naïve-B-cells found in N5 and N7, and neutrophils, in N8 (See Fig. 3 and Table 1).

### VAF_RNA_ estimation

VAF_RNA_ is assessed from the individual alignments as we have previously described [28], using the high quality heterozygous SNV sites as inputs for ReadCounts [22]. At each position of interest, ReadCounts estimates the number of sequencing reads harboring the variant and the reference nucleotide (n_var_ and n_ref_, respectively), calculates VAF_RNA_ (VAF_RNA_ = n_var_ / (n_var_ + n_ref_), and filters out positions not covered by the user-defined minimum number of reads (minR); minR is constant across the genome [22]. For the herein presented analysis, we used minR> 10. To qualify for scReQTL, a variant is required to have variable VAF_RNA_ from a minimum of 20 cells from the same cell type (per donor). The VAF_RNA_ distribution is then examined and loci with non-variable VAF_RNA_ are filtered out. Loci were considered non-variable if: (1) over 75% of the VAF_RNA_ values are in the range of 0.5 ± 0.1 (corresponding to stable biallelic expression), and (2) over 75% of the VAF_RNA_ values are in the ranges 0–0.25 or 0.75–1 (corresponding to predominantly monoallelic or skewed allelic expression).

### ScReQTL computations

*SNV-GE correlations* (scReQTLs) were computed for each donor, across the cells of each type separately. To qualify for scReQTLs analysis, an SNV locus is required to have informative and variable VAF_RNA_ estimations (minR> 10) from at least 20 cells per analysis. The variable VAF_RNA_ were correlated to the normalized GE values of the most variable genes using a linear regression model as implemented in Matrix eQTL [17]. The top 15 principal components of the GE were used as covariates (Supplementary Figure 16). Cis and trans correlations were annotated as previously described for the bulk ReQTLs [25]. Briefly, because scReQTLs are assessed from transcripts, we assign cis-correlation based on the co-location of the SNV locus within the transcribed gene, using the gene coordinates [50]. All the scReQTLs including SNVs residing in genes different from the expression-correlated genes are annotated as trans-scReQTLs.

### Statistical analyses

Throughout the analysis we used the default statistical tests (with built-in multiple testing corrections) implemented in the used software packages (Seurat, SingleR, Matrix eQTL), where *p*-value of 0.05 was considered significant, unless otherwise stated. For estimation of significant scReQTL, we applied FDR as implemented in the Matrix eQTL package. Specifically, once Matrix eQTL discovers a set of significant gene-SNP pairs, it estimates a corresponding q-value (FDR) for each of them using Benjamini–Hochberg procedure under the assumption that the tests are independent or positively correlated [17, 51]. For estimation of differences in overlap between scReQTL SNVs, GWAS and ClinVar, chi-square test was used. For assessment of enrichment of scReQTLs in known gene-gene interactions, a permutation test with 10,000 permutations was applied on the findings at minR = 10, and with 1000 permutations for the scReQTL analyses at minR< 10. For each permutation, a random set of gene-gene pairs of the same size as the observed data was selected. The p-value was defined as the fraction of permutations in which the number of gene-gene pairs found in the known interaction database was at least as great as the number found in the observed data.

## Supplementary Information

**Additional file 1.**

**Additional file 2.**

**Additional file 3.**

**Additional file 4.**

**Additional file 5.**

**Additional file 6.**

**Additional file 7.**

## Data Availability

All data generated or analyzed during this study are included in this published article and its supplementary information files.
