# Supplementary material for: scReQTL: an approach to correlate SNVs to gene expression from individual scRNA-seq datasets
Source: BMC Genomics. 2021 Jan 8;22:40. doi: 10.1186/s12864-020-07334-y (PMC7791999; doi:10.1186/s12864-020-07334-y)
Supplement: Supplementary file 1 — Additional file 1. [file 12864_2020_7334_MOESM1_ESM.pdf]

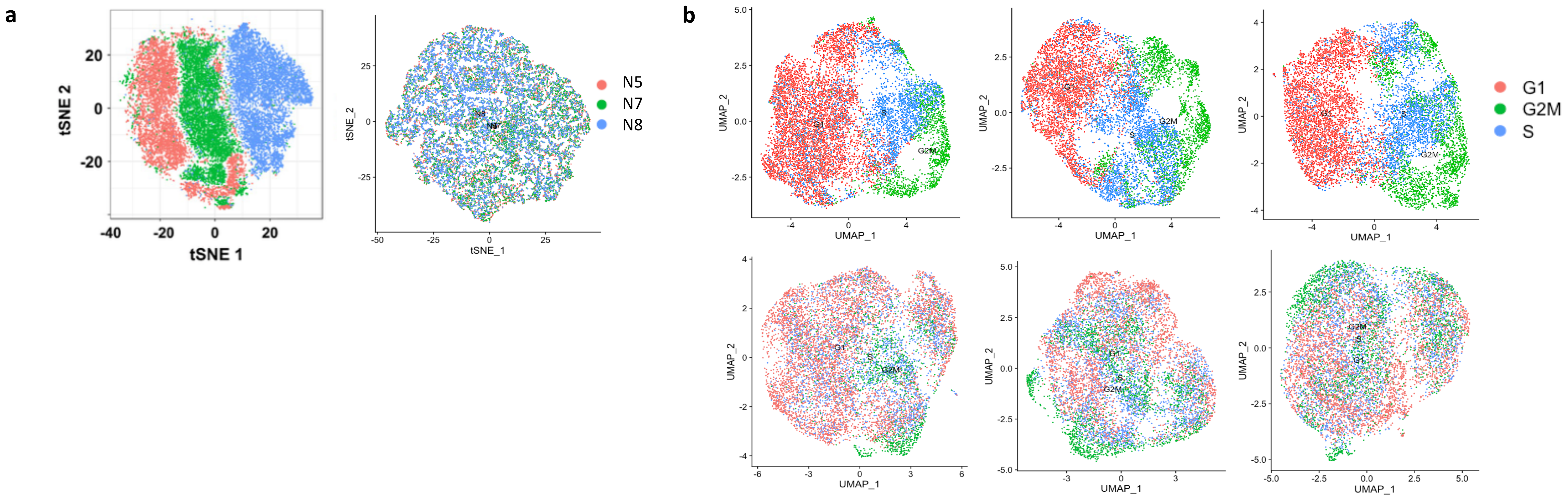

Supplementary Figure 1. a) t-SNE plots before (left) and after (right) correction for batch effects using Seurat in the three samples (from left to right N8, N7, N5). Strong batch effects are visible before the correction. **b**) **Top**: cell cycle scores based on expression of G2/M and S phase markers assigned using Seurat (from left to right N8, N7, N5). Bottom: Scores after regressing out the cell cycle source of heterogeneity.

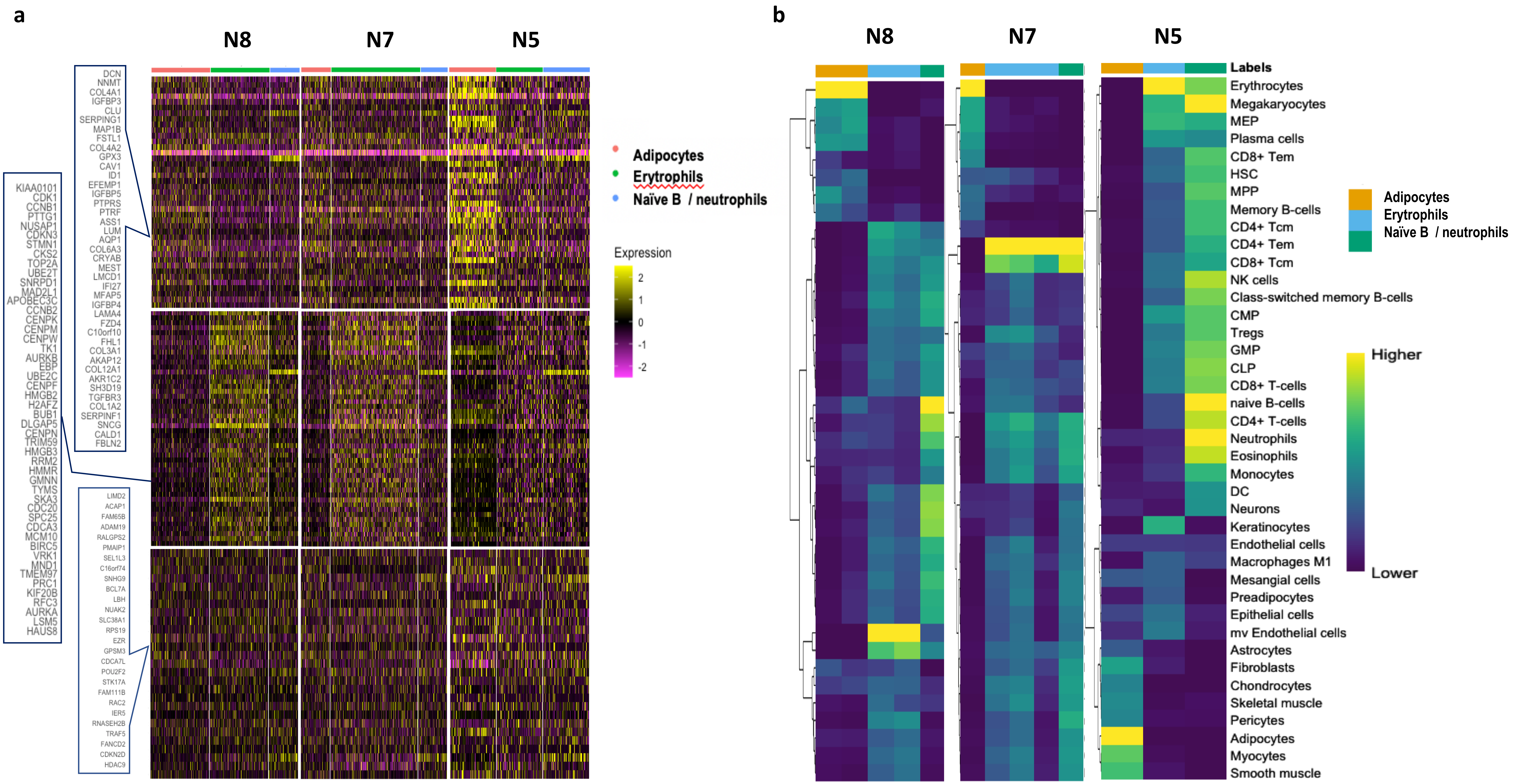

Supplementary Figure 3. a. Heatmap showing the gene-lists retained as those representing the most similar to the reference cell-type expression patterns. **b**. Heatmaps of SingleR scores for top correlated cell types from each of Seurat generated clusters. SingleR uses expression data to regenerate the clusters, and for each cluster, calculates the Spearman coefficient for the genes in the reference dataset. Then, it uses multiple correlation coefficient to collect a single value per cell type per cluster.

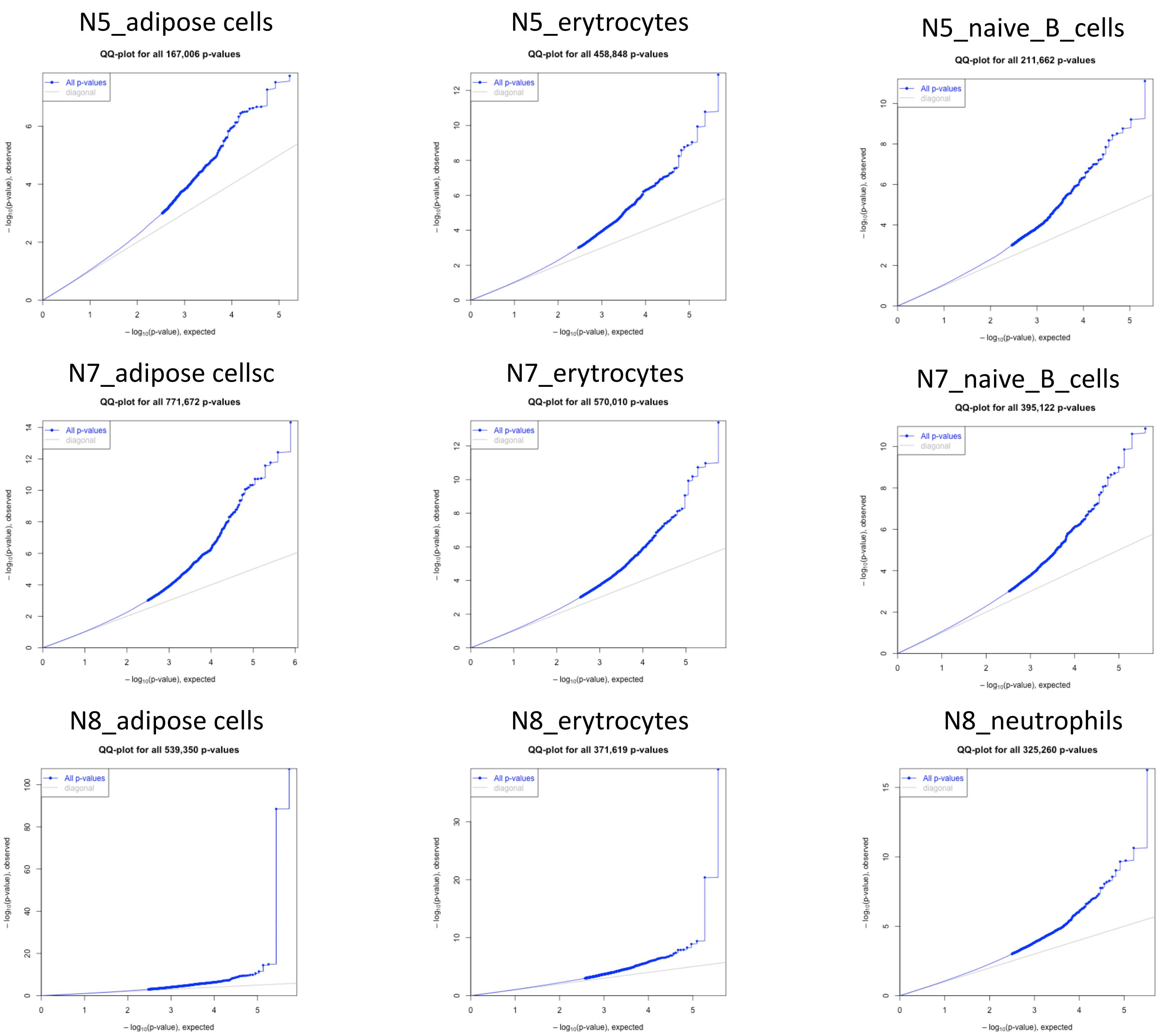

Supplementary Figure 3. QQ-plots of the scReQTL P-values.

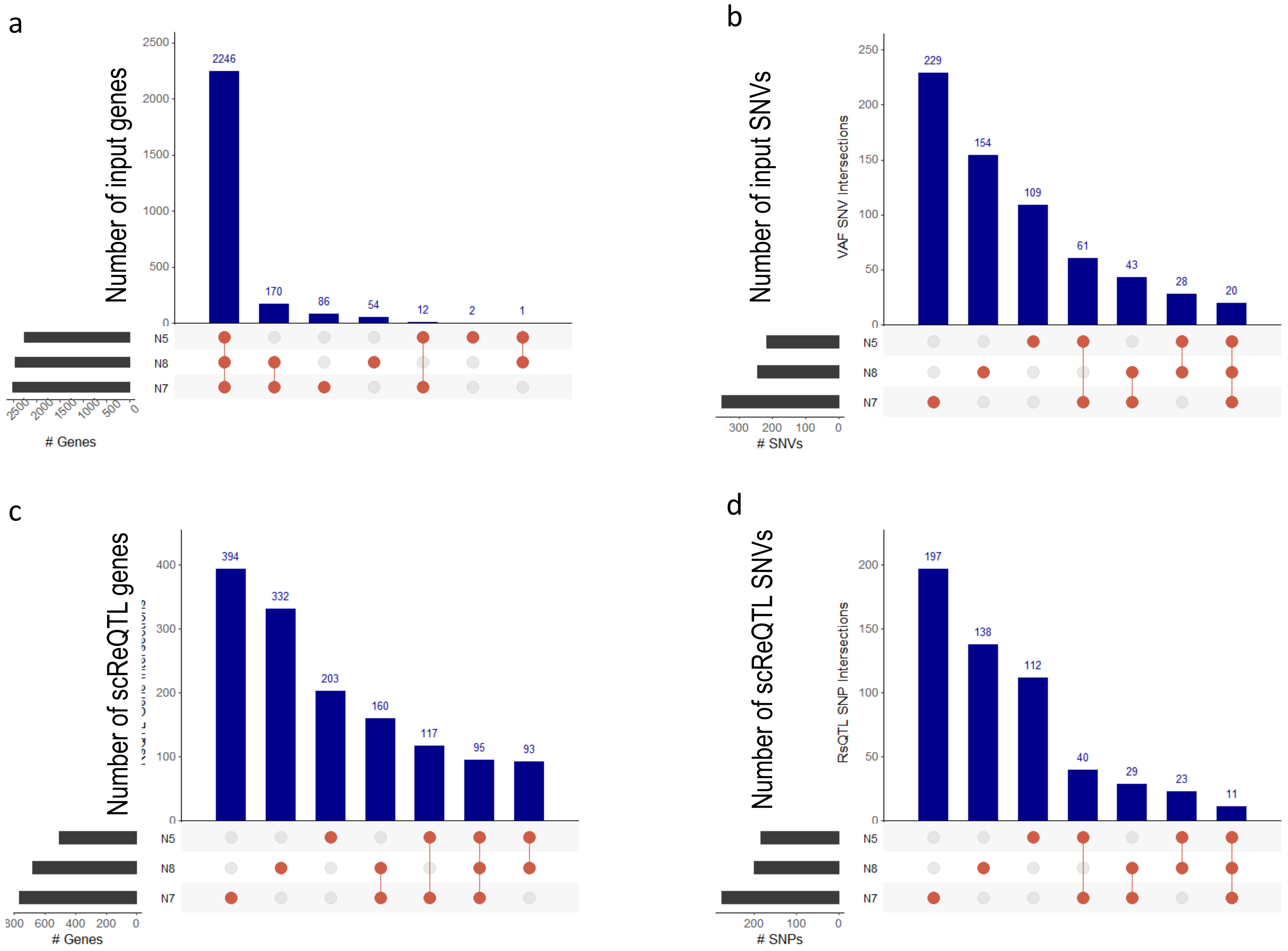

Supplementary Figure 4. Relative representation of donor-specific and shared ScReQTL genes and SNVs. **a**) Input genes for scReQTL analysis were shared between the three donors to a large extend. **b**) Input SNV sites for scReQTL analysis were largely donor-specific, with only 20 SNVs shared across the 3 donors. **c**) Shared and donor-specific genes participating in significant scReQTLs. **d**) Shared and donor-specific SNVs participating in significant scReQTLs; 11 of the 20 shared input SNVs participated in scReQTLs shared across the 3 donors.

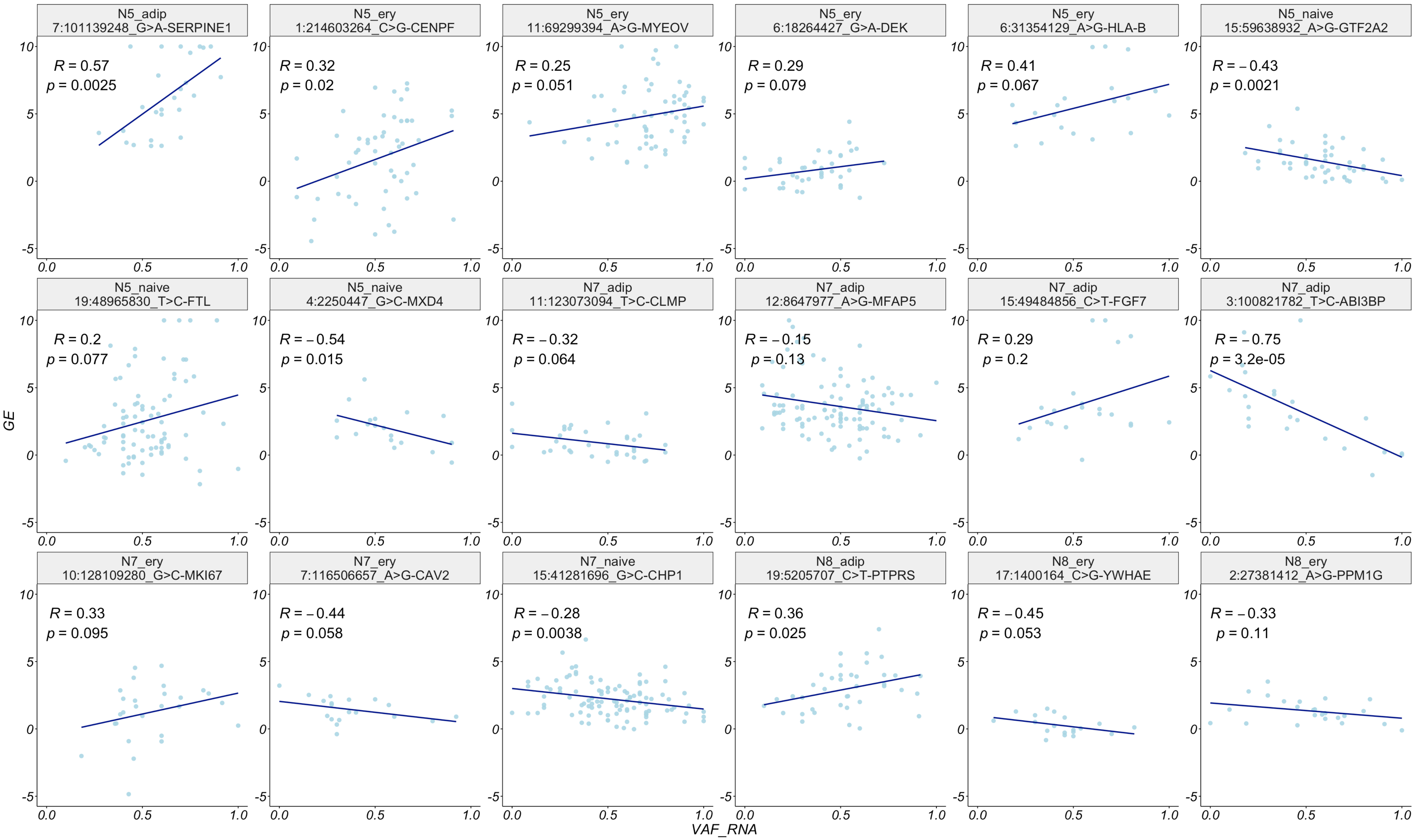

Supplementary Figure 5. Examples of scReQTL correlations between SNVs and their harboring genes (cis-scReQTLs) at FDR=0.1. Note that the displayed P-values are calculated based on the input for the plots generated using the R-package ggplot2 and do not represent the FDR—corrected values from the scReQTL analysis performed with Matrix eQTL.

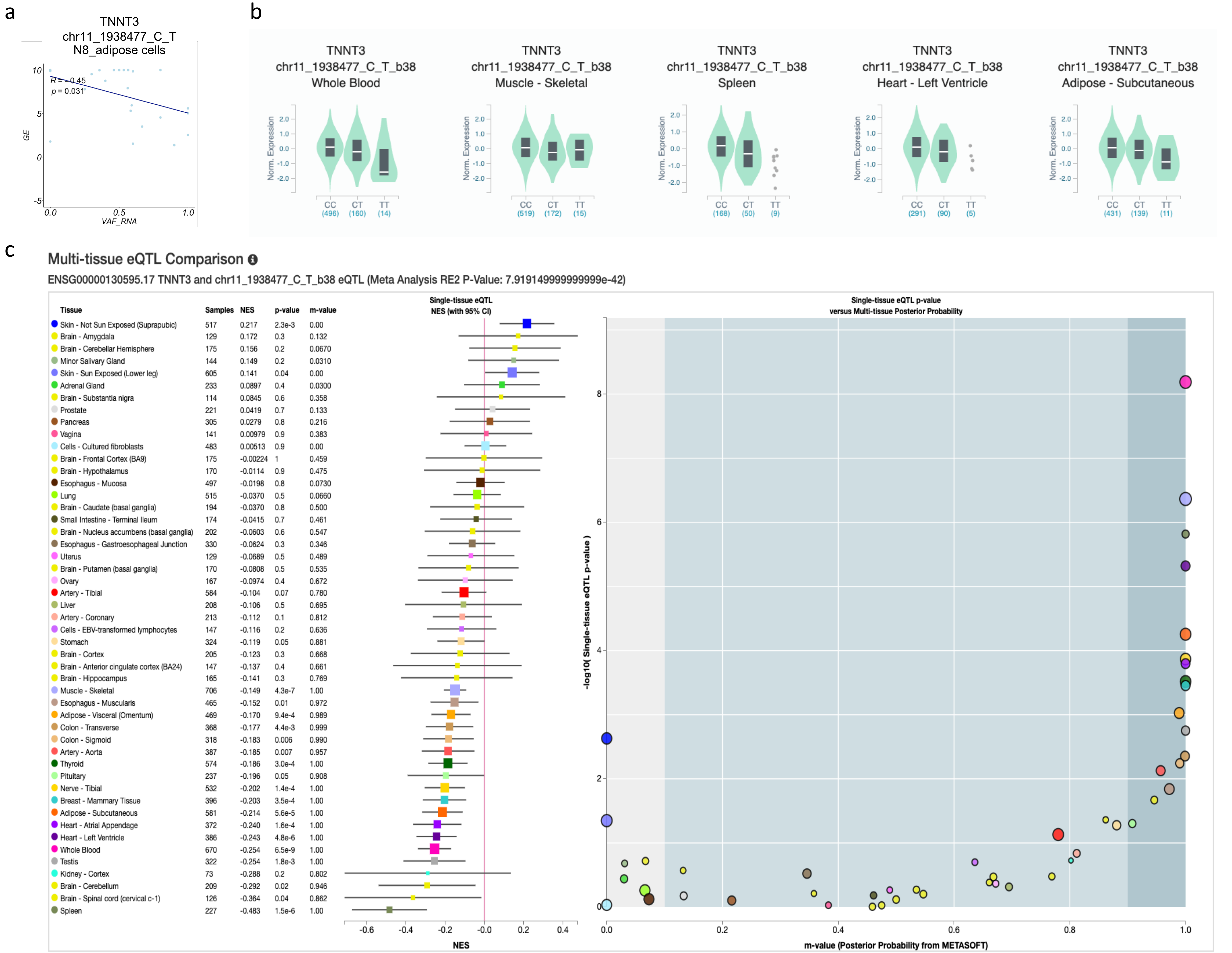

Supplementary Figure 6. scReQTL and eQTLs between the SNV at 11:1938477_C>T and its harboring gene *TNNT3* (cis-scReQTL). **a**) scReQTL between the SNV at 11:1938477_C>T and *TNNT3*. **b**) eQTLs between the SNV at 11:1938477_C>T and *TNNT3* reported in the GTEX database in 5 different tissues. In all 5 tissues, the eQTLs were consistent in terms of directionality (negative). **c**) Multi-tissue comparisons of the eQTL 11:1938477_C>T - *TNNT3*.

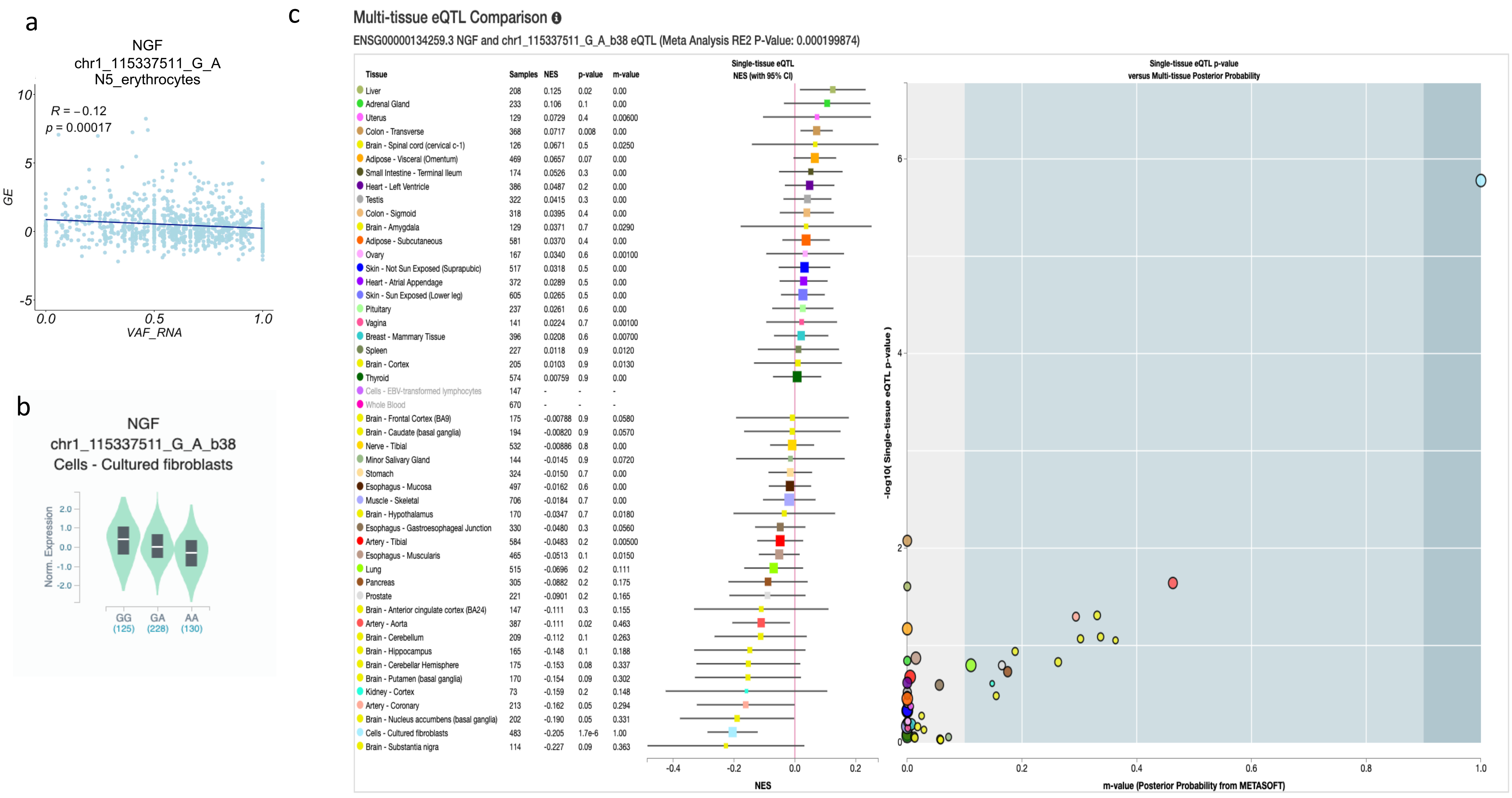

Supplementary Figure 7. scReQTL and eQTLs between the SNV at 1:115337511_G>A and the gene *NGF* (trans-scReQTL). **a**) scReQTL between the SNV at 1:115337511_G>A and the gene *NGF*. **b**) eQTL between the SNV at 1:115337511_G>A and the gene *NGF* reported in the GTEX in cultured fibroblasts; the scReQTL and the eQTL were consistent in terms of directionality (negative). **c**) Multi-tissue comparisons of the eQTL 1:115337511_G>A - *NGF*.

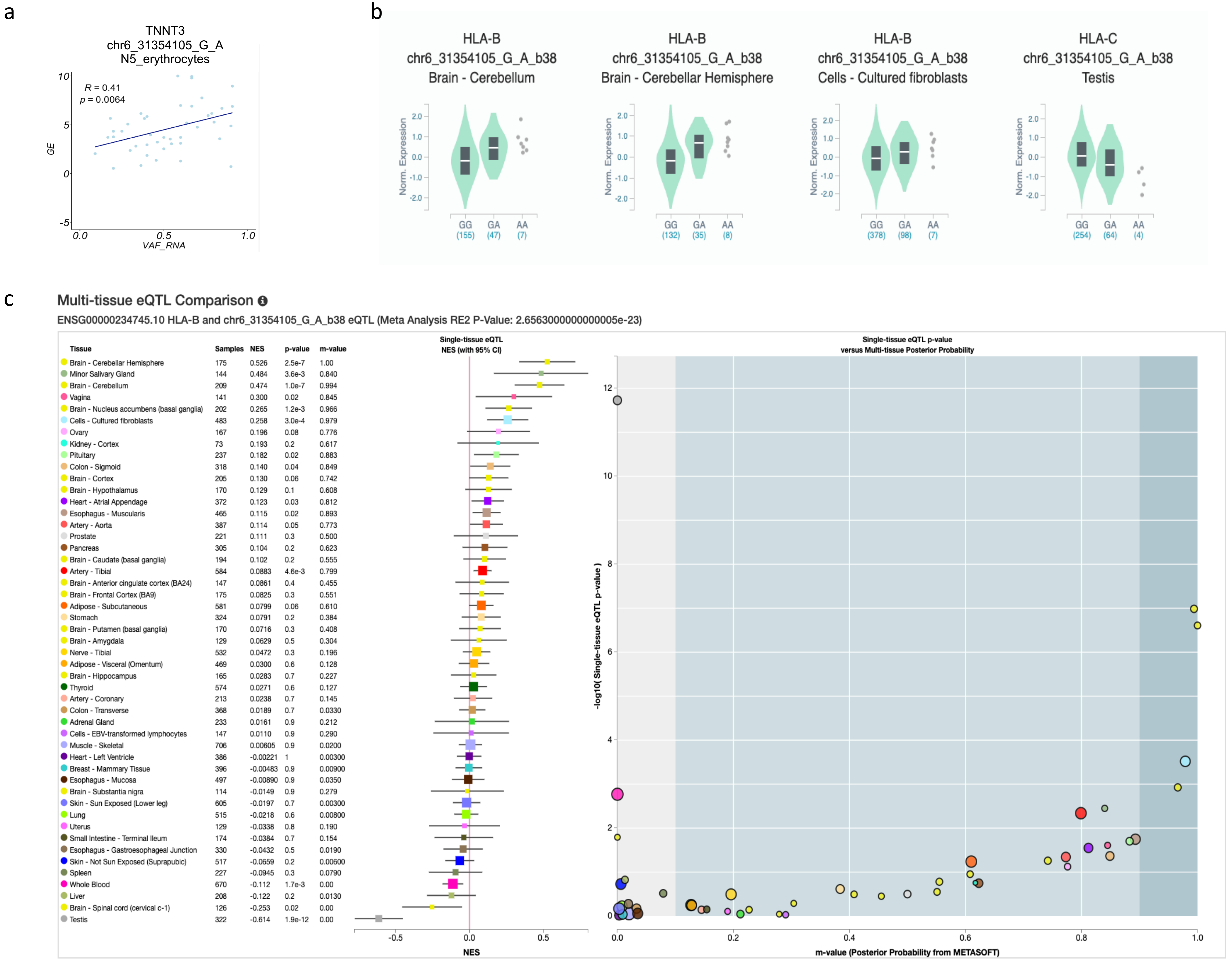

Supplementary Figure 8. scReQTL and eQTLs between the SNV at 6:31354105_G>A and its harboring gene *HLA-B* (cis-scReQTL). **a**) scReQTL between the SNV at 6:31354105_G>A and *HLA-B*. **b**) eQTLs between the SNV 6:31354105_G>A and HLA-B reported in the GTEX in 4 tissues; the eQTLs correlations were positive in three of the tissues (and agreed with the scReQTL), but in the opposite direction with the eQTL is 1 tissue (testis). **c**) Multi-tissue comparisons of the eQTL at 6:31354105_G>A - *HLA-B*.

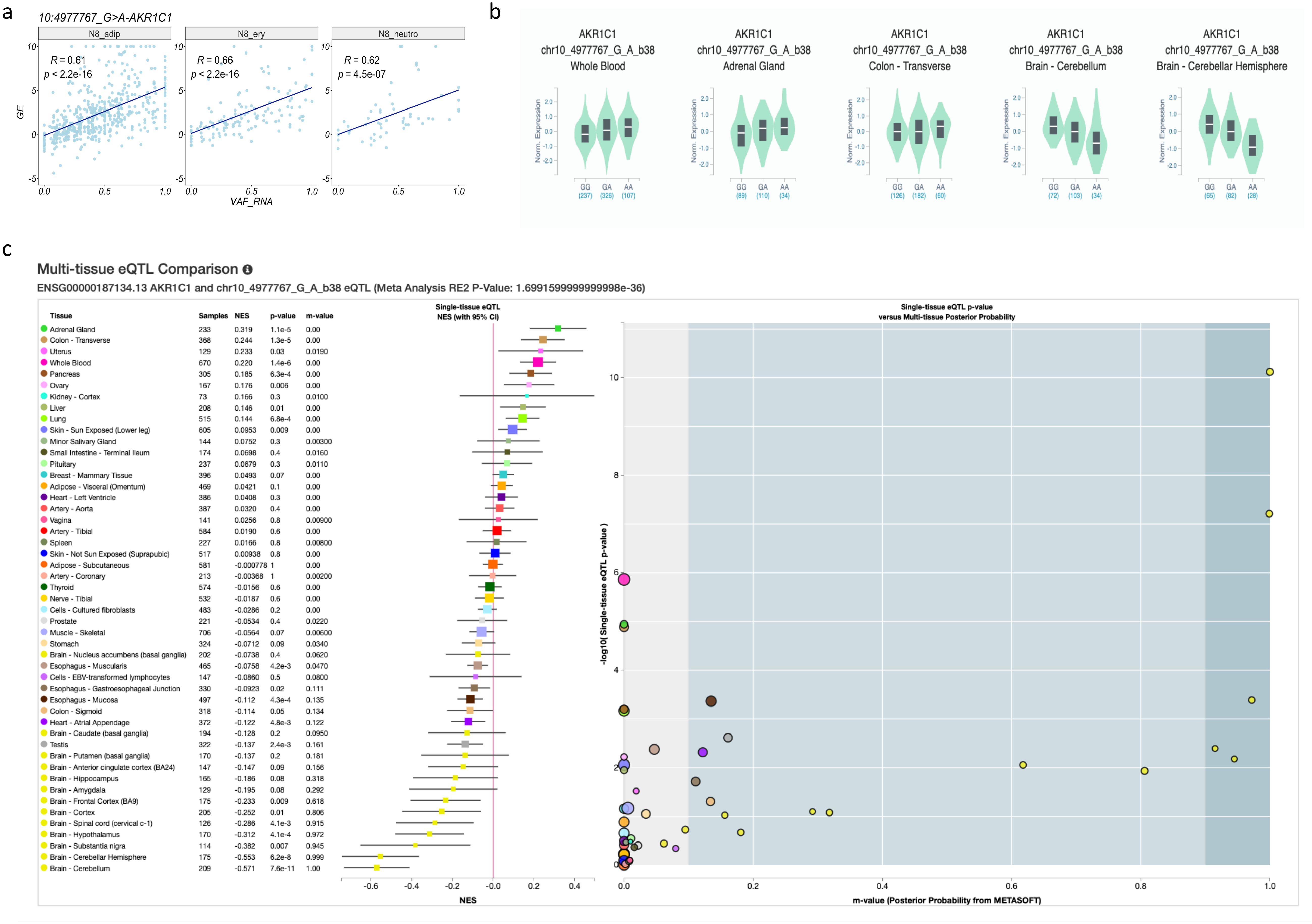

Supplementary Figure 9. scReQTL and eQTLs between the SNV at 10:4977767_G>A and its harboring gene *AKR1C1* (cis-scReQTL). a) scReQTL between the SNV at 10:4977767_G>A and *AKR1C1*. b) eQTLs between the SNV 10:4977767_G>A and *AKR1C1* reported in the GTEX in 5 tissues; the eQTLs correlations were positive in three of the tissues (and agreed with the scReQTL), but in the opposite direction with the eQTL is 2 tissues. c) Multi-tissue comparisons of the eQTL at 10:4977767_G>A and *AKR1C1*.

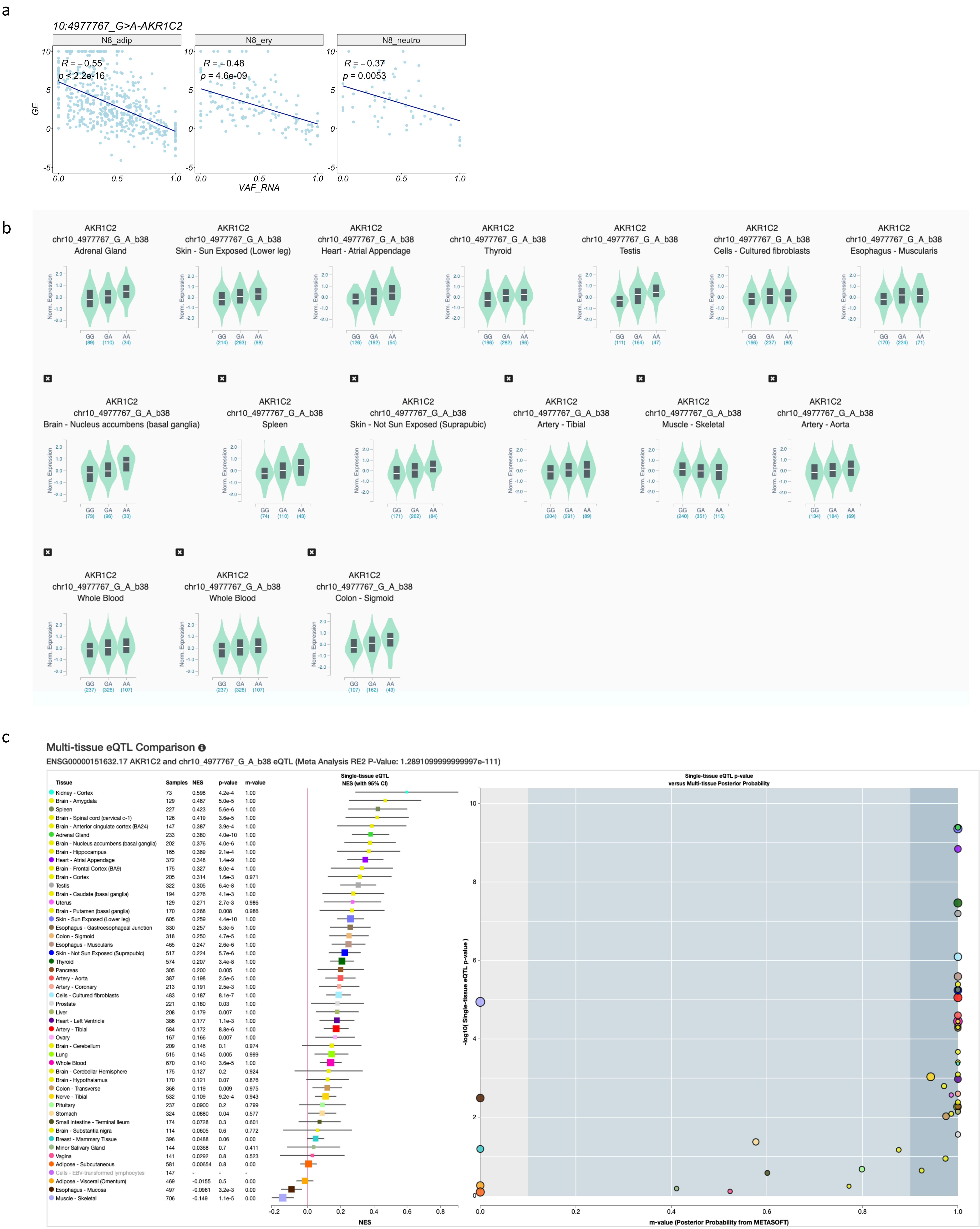

Supplementary Figure 10. scReQTL and eQTLs between the SNV at 10:4977767_G>A and *AKR1C2* (trans-scReQTL). a) scReQTL between the SNV at 10:4977767_G>A and *AKR1C2*. b) eQTLs between the SNV 10:4977767_G>A and *AKR1C2* reported in the GTEX in 6 tissues; the eQTLs correlations were positive in 15 of the tissues, but negative in 1 – skeletal muscle – where they agreed with the scReQTL. c) Multi-tissue comparisons of the eQTL 10:4977767_G>A - *AKR1C2* .

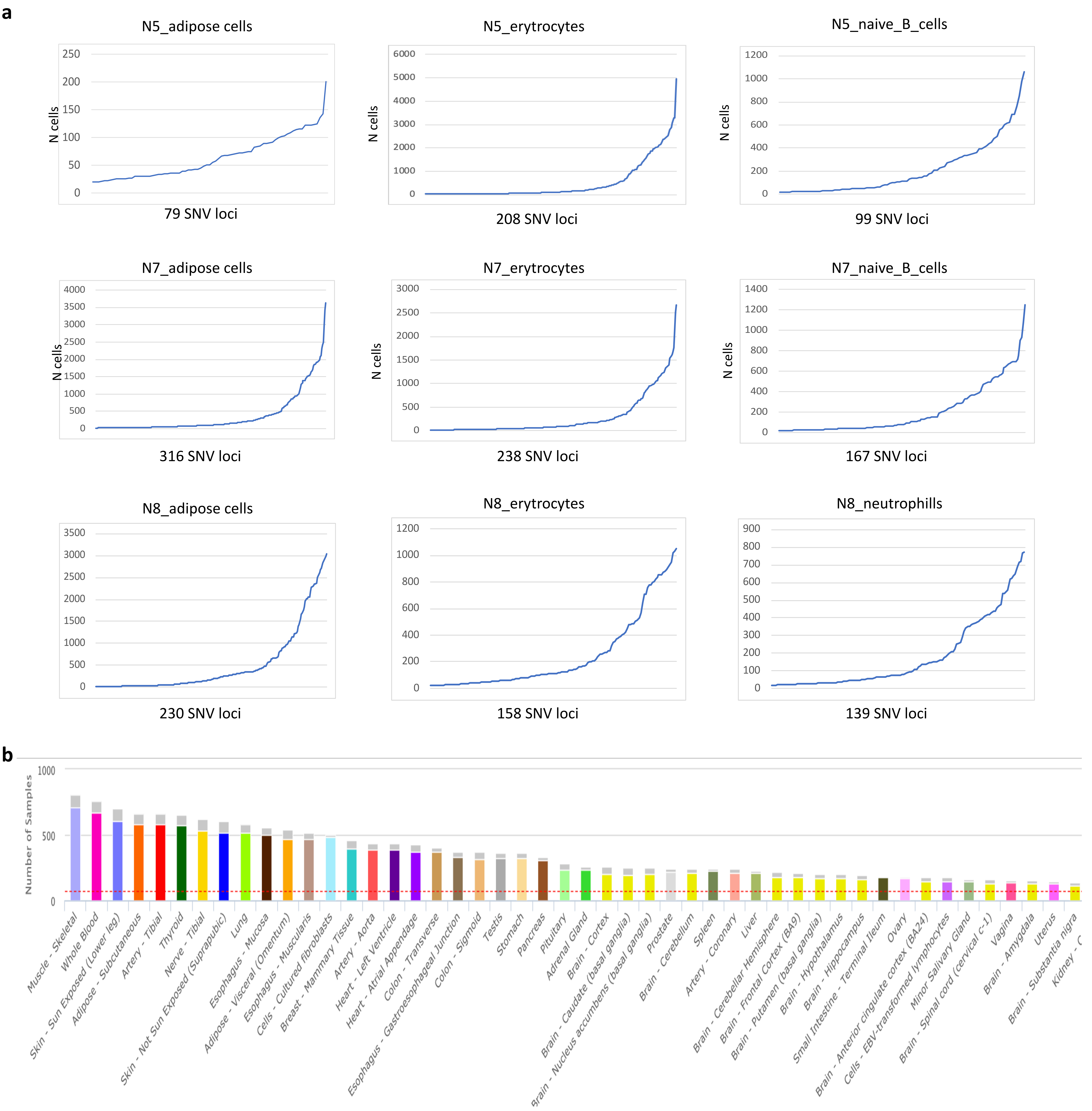

Supplementary Figure 11. Number of input cells for scReQTL and input samples (individuals) eQTL in GTEX. **a**) Number of cells with informative VAF_RNA values for scReQTL analysis per donor stratified by cell type. **b**) Number of individuals with genotypes for eQTL analyses in GTEx.

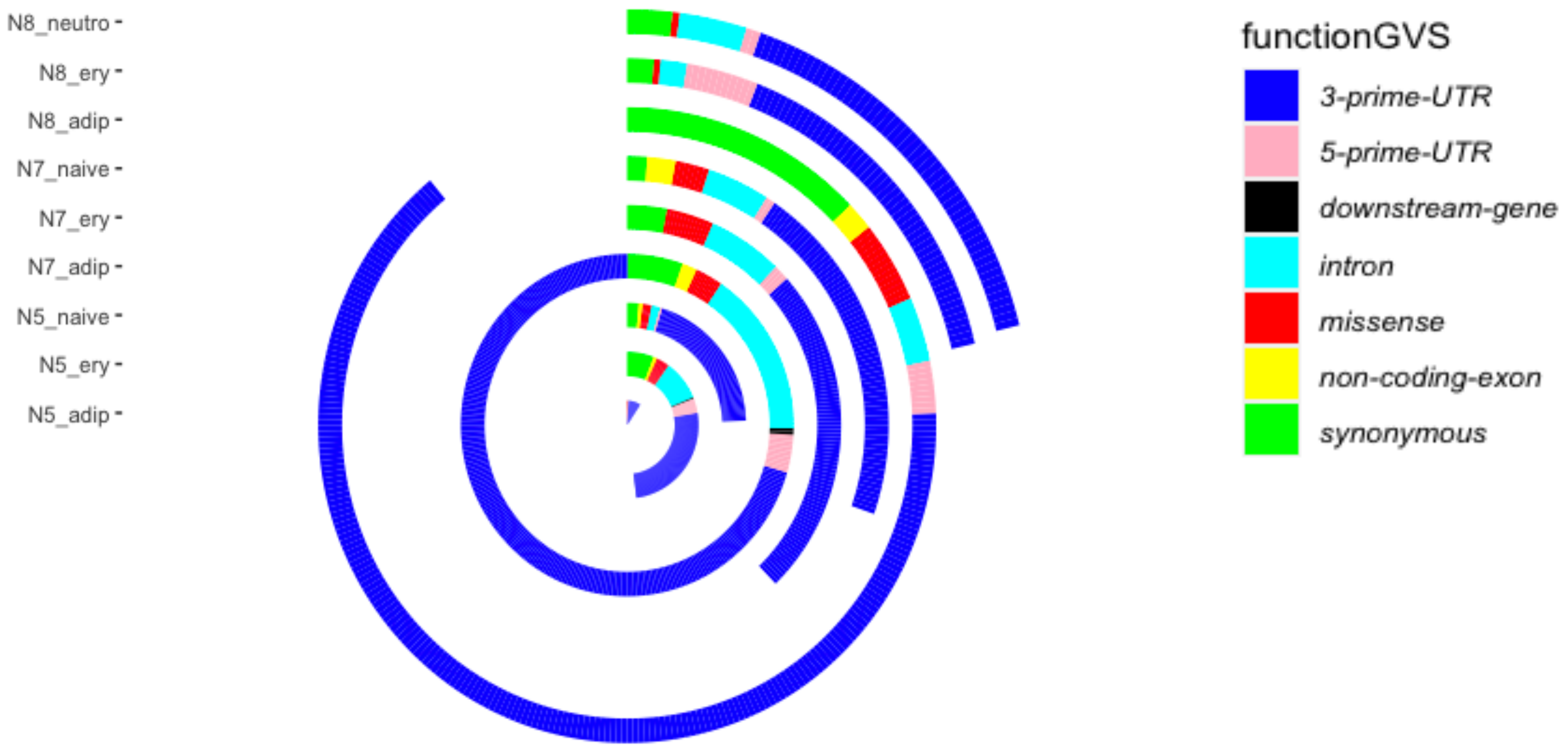

Supplementary Figure 12. The majority of the scReQTL SNVs resided in the 3'UTR of their harboring gene (70.2%), followed by exonic SNVs (16.2%), and intronic SNVs (11.2%)

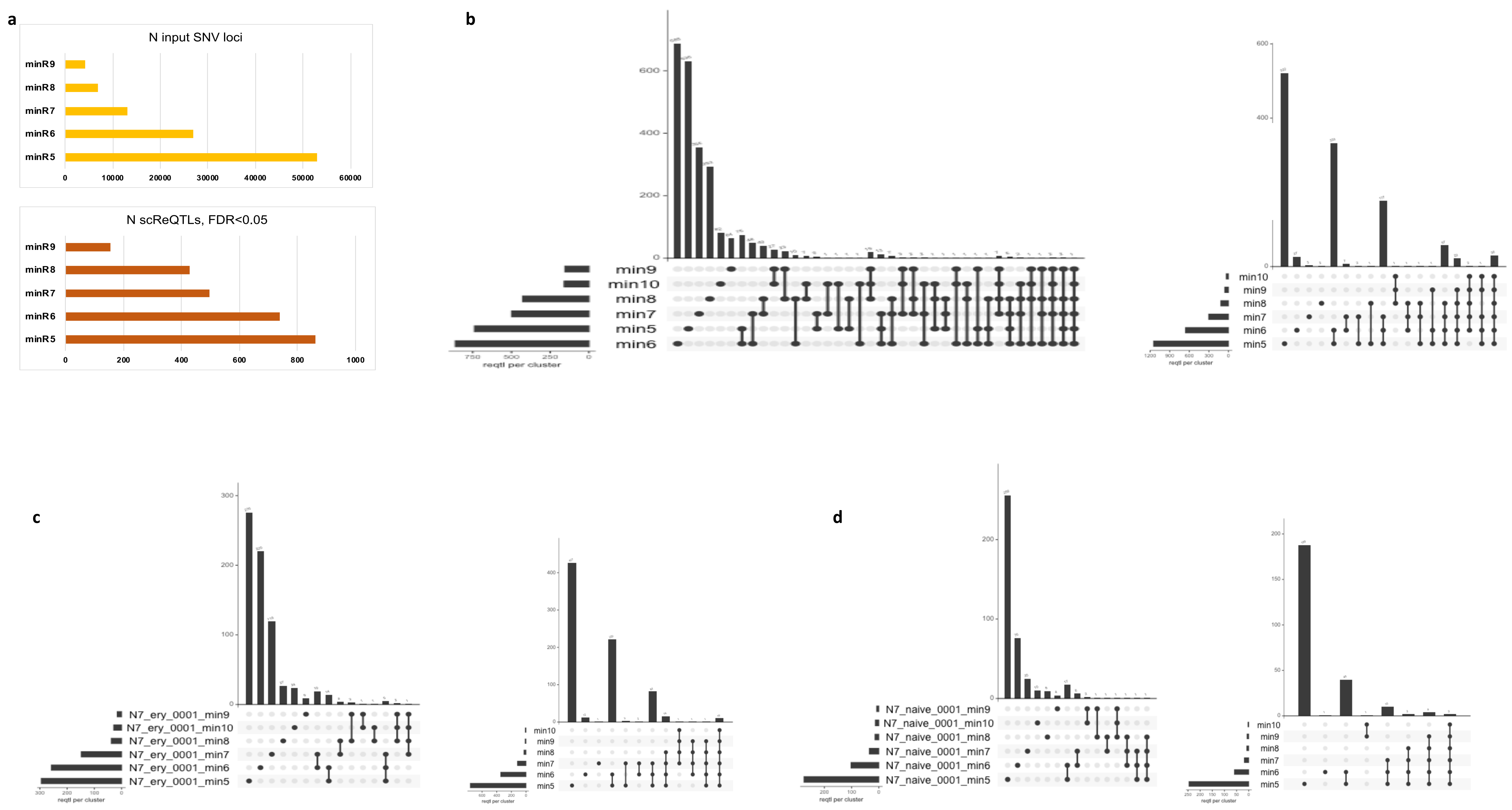

Supplementary Figure 13. **a.** Number of input SNV loci (top) and significant scReQTL with minR between 5 and 9 (Sample N7_adip). **b-d**) Shared and distinct scReQTLs (left) and input SNV loci (right) at different minR cut-offs, minR = 10,9,8,7,6 and 5 unique sequencing reads are analyzed (Sample N7_adip (b), N7_ery (c) and N7_naive (d). Both input SNV loci and scReQTLs show partial overlap across the different minR cut-offs.

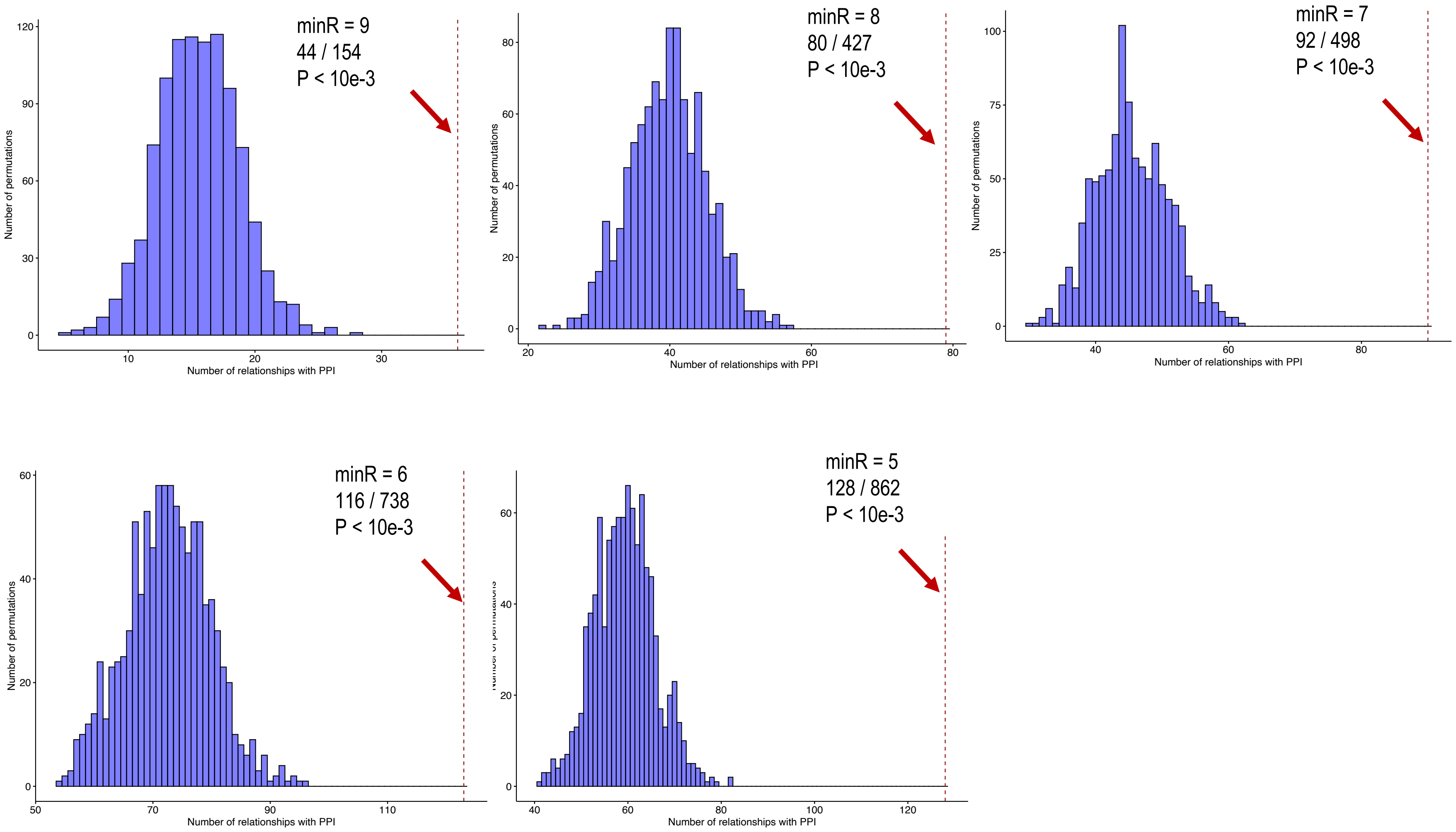

Supplementary Figure 14. Permutation test for assessment of enrichment of trans scReQTLs at different minR in known gene-gene interactions obtained from the STRING database; 1000 permutations were used. The p-value (p<10e-3) was defined as the fraction of permutations in which the number of gene-gene pairs found in the known interaction database was at least as great as the number found in the observed data. This analysis showed significant enrichment with known gene-gene interactions.

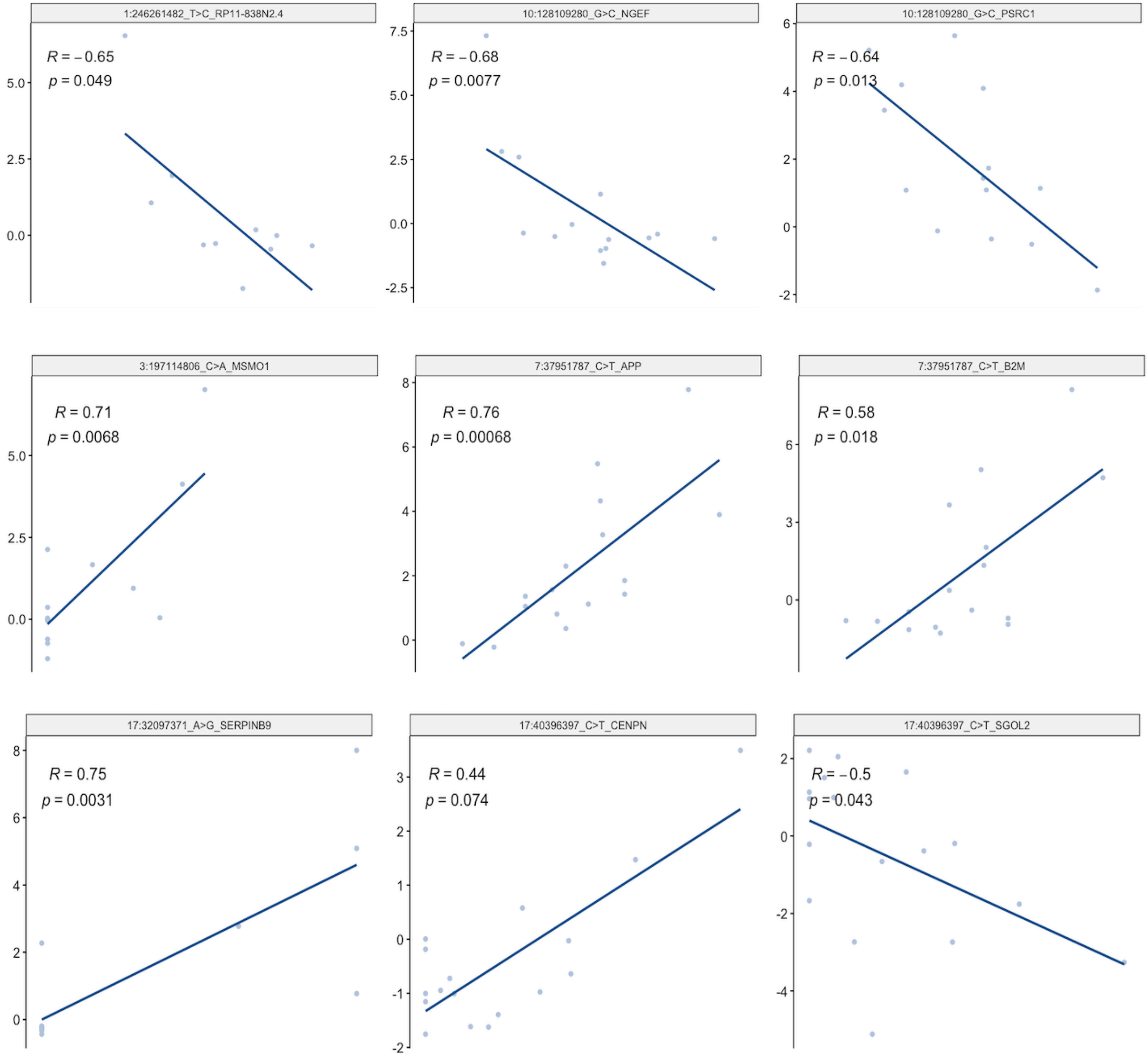

Supplementary Figure 15. scReQTLs estimated from satisfying the minR in10-20 cells, sample N7_adip.

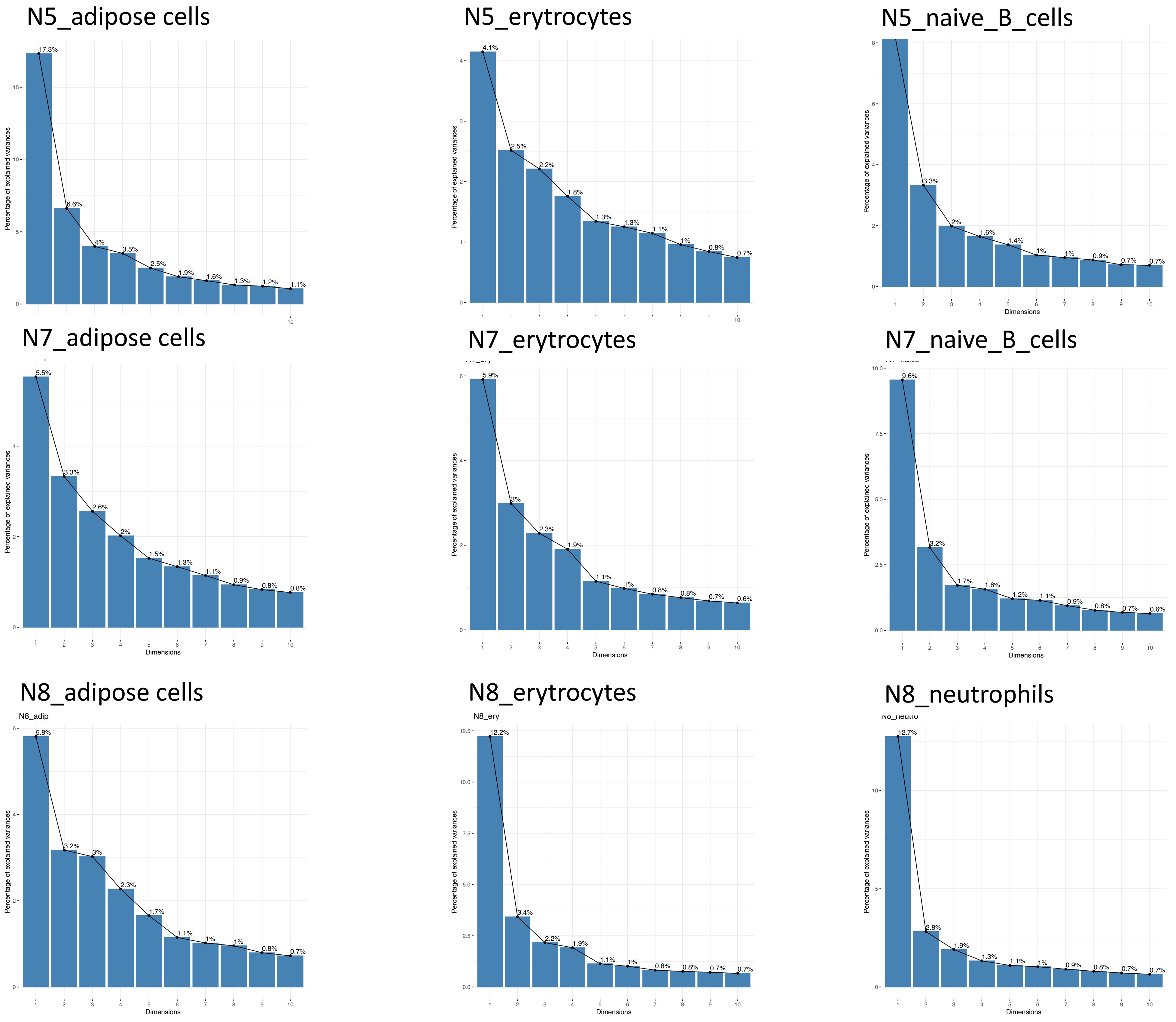

Supplementary Figure 16. Percentage explained variance of the top 10 Principal Components (PC) for gene expression.
